# Thio-2 inhibits key signaling pathways required for the development and progression of castration resistant prostate cancer

**DOI:** 10.1158/1535-7163.MCT-23-0354

**Published:** 2024-02-27

**Authors:** Antje Neeb, Ines Figueiredo, Denisa Bogdan, Laura Cato, Jutta Stober, Juan M. Jiménez-Vacas, Victor Gourain, Irene I. Lee, Rebecca Seeger, Claudia Muhle-Goll, Bora Gurel, Jonathan Welti, Daniel Nava Rodrigues, Jan Rekowski, Xintao Qiu, Yija Jiang, Patrizio Di Micco, Borja Mateos, Stasė Bielskutė, Ruth Riisnaes, Ana Ferreira, Susana Miranda, Mateus Crespo, Lorenzo Buroni, Jian Ning, Suzanne Carreira, Stefan Bräse, Nicole Jung, Simone Gräßle, Amanda Swain, Xavier Salvatella, Stephen R. Plymate, Bissan Al-Lazikani, Henry W. Long, Wei Yuan, Myles Brown, Andrew C. B. Cato, Johann S. de Bono, Adam Sharp

**Affiliations:** 1 Institute of Cancer Research, London, UK; 2 Dana-Farber Cancer Institute, Boston, US; 3 Karlsruhe Institute of Technology (KIT), Institute for Biological and Chemical Systems – Biological Information Processing (IBCS-BIP); 4 Karlsruhe Institute of Technology (KIT), Institute for Biological Interfaces 4 (IBG-4); 5 MD Anderson Cancer Centre, Texas, US; 6 Institute for Research in Biomedicine, The Barcelona Institute of Science and Technology, Barcelona, Spain; 7 Karlsruhe Institute of Technology (KIT), Institute of Biological and Chemical Systems – Functional Molecular Systems (IBCS-FMS); 8 Catalan Institution for Research and Advanced Studies, Barcelona, Spain; 9 University of Washington, Seattle, Washington, USA; 10 Geriatrics Research, Education and Clinical Center, VAPSHCS, Seattle, Washington; 11 Royal Marsden NHS Foundation Trust, London, UK

## Abstract

Therapies that abrogate persistent androgen receptor (AR) signaling in castration resistant prostate cancer (CRPC) remain an unmet clinical need. The N-terminal domain (NTD) of the AR that drives transcriptional activity in CRPC remains a challenging therapeutic target. Herein we demonstrate that BAG-1 mRNA is highly expressed and associates with signaling pathways, including AR signaling, that are implicated in the development and progression of CRPC. In addition, interrogation of geometric and physiochemical properties of the BAG domain of BAG-1 isoforms identifies it to be a tractable but challenging drug target. Furthermore, through BAG-1 isoform mouse knockout studies we confirm that BAG-1 isoforms regulate hormone physiology and that therapies targeting the BAG domain will be associated with limited ‘on-target’ toxicity. Importantly, the postulated inhibitor of BAG-1 isoforms, Thio-2, suppressed AR signaling and other important pathways implicated in the development and progression of CRPC to reduce the growth of treatment resistant prostate cancer cell lines and patient derived models. However, the mechanism by which Thio-2 elicits the observed phenotype needs further elucidation since the genomic abrogation of BAG-1 isoforms was unable to recapitulate the Thio-2 mediated phenotype. Overall, these data support the interrogation of related compounds with improved drug-like properties as a novel therapeutic approach in CRPC, and further highlight the clinical potential of treatments that block persistent AR signaling which are currently undergoing clinical evaluation in CRPC.

## Introduction

Prostate cancer is the most commonly diagnosed non-cutaneous malignancy in men and is a leading cause of male mortality (1). Despite the development of novel hormonal therapies targeting the androgen receptor (AR), such as abiraterone, enzalutamide, apalutamide and darolutamide, that have improved the outcome for patients with advanced castration sensitive prostate cancer (CSPC) and castration resistant prostate cancer (CRPC), primary and secondary resistance to therapy remains inevitable (2, 3). Treatment resistance is driven, in part, by persistent AR signaling associated with the emergence of AR amplification, AR activating mutations, and constitutively active AR splice variants (3–7). The development of novel therapies that block persistent AR signaling is an urgent unmet clinical need.

One attractive therapeutic strategy is to target molecular co-chaperones, such as BAG-1 (BCL-2-associated athanogene-1), that have been reported to bind and enhance AR activity. BAG-1 interacts with a wide range of molecular targets to regulate multiple cellular pathways (including apoptosis, proliferation, metastasis, and nuclear hormone receptor transactivation) important for the development and progression of cancer (8–10). Three major isoforms, BAG-1L (50kDa), BAG-1M (46kDa) and BAG-1S (36kDa), exist in humans and are generated through alternative initiation of translation from a single mRNA (11). Consistent with this, BAG-1L has a unique N-terminus which contains a nuclear localization sequence and is predominantly localized within the nucleus, supporting its interaction with the AR, whereas the other isoforms (BAG-1M and BAG-1S) are found in both the nucleus and cytoplasm (8–10). All BAG-1 isoforms share a common C-terminus, which contains the highly conserved BAG domain, critical for the interaction between BAG-1 isoforms and the heat shock chaperones, HSC70/HSP70 (12–14). Importantly, the BAG-1:HSC70/HSP70 interaction is reported to be critical for BAG-1 function and therefore therapies targeting this interaction are an attractive strategy to overcome BAG-1 function in cancer (8–10, 15–22).

BAG-1L plays a critical role in transactivation of the AR and nuclear BAG-1 protein expression correlates with important clinical characteristics in prostate cancer (15–18, 23–25). Through its C-terminal BAG domain, BAG-1L binds to the AR NTD, leading to receptor transactivation (15–18). Consistent with this, loss of BAG-1L abrogates AR signaling and reduces prostate cancer growth (15). In addition, expression of nuclear BAG-1 correlates with worse outcome from AR targeting therapies in patients with CRPC (15). Moreover, mutagenesis studies demonstrated that specific amino acid residues within the BAG domain of BAG-1L are critical for the BAG-1L:AR interaction and AR transactivation (15). Finally, Thio-2, a tool compound that has been predicted to bind the BAG domain within BAG-1 isoforms through *in-silico* docking experiments, and more recently A4B17 and X15695, have been reported to inhibit BAG-1L-mediated AR NTD transactivation (15, 26–29). Taken together, these data support targeting the BAG domain of BAG-1 isoforms as an attractive therapeutic strategy to overcome persistent AR signaling in CRPC.

Herein we confirm that BAG-1 mRNA is highly expressed and associates with signaling pathways critical for the development and progression of CRPC. In addition, we report that the BAG domain provides a tractable drug target, and that BAG-1 mouse knockout studies indicate that BAG-1 isoforms may mediate hormone physiology and targeting the BAG domain should be associated with minimal ‘on-target’ toxicity. Moreover, we show that Thio-2 which is predicted to bind the BAG domain, suppresses AR activity and other key signaling pathways, to inhibit the growth of prostate cancer cell lines and patient derived models. The Thio-2 phenotype was not copied by either BAG-1 isoform knockdown or knockout, suggesting, that in these studies, the mechanism of action of Thio-2 was not mediated through BAG-1 isoforms. Taken together, these data support the interrogation of related compounds, with improved drug-like properties, as a novel therapeutic strategy for CRPC.

## Materials and Methods

### CRPC patient transcriptome analyses

CRPC transcriptomes from the Stand Up To Cancer/Prostate Cancer Foundation (SU2C/PCF) cohort were downloaded and re-analyzed (30, 31). CRPC transcriptomes from the Institute of Cancer Research/Royal Marsden Hospital (ICR/RMH) cohort were reanalyzed (32). Paired-end transcriptome sequencing reads for each of the SU2C/PCF (n = 159) and ICR/RMH (n = 95) cohorts were aligned to the human reference genome (GRCh37/hg19) using Tophat2 (v2.0.7). Gene expression, Fragments Per Kilobase of transcript per Million mapped reads (FPKM), was calculated using Cufflinks. The top expressed genes (n = 15000) were analyzed for each cohort respectively. Gene Set Enrichment Analysis (GSEA) was performed using the pre-Ranked algorithm from GSEA software (v4.1.0). The top genes were ranked from high to low using the Spearman correlation coefficient between each gene’s expression (FPKM) and BAG-1 expression (FPKM), and subsequently used in analysis. Results were obtained using Molecular Signatures Database hallmark gene collection (33).

### canSAR platform

Briefly, the algorithm identifies up to 10 cavities on a 3D-structure and measures ~30 geometric and physicochemical properties for each of these cavities to determine ligandability. The tools and methodologies used are available at our online canSAR platform (34–36). Since proteins are mobile, and this mobility affects the formation of druggable cavities, we performed Monte Carlo simulations to explore limited movements of each structure. The simulations were performed using the CONCOORD method (37). Yamber2, Van der Waals and CONCOORD default bond/angle were set as parameters. A total of 449 alternative structures were shaped (at least 10 structures for each of the 44 original structures, Supplementary Table 1) and all cavities identified were assessed for ligandability by canSAR algorithm as described above.

### BAG-1 exon 1 and exon 2 knockout mice

Studies with BAG-1 knockout mice were performed at the Karlsruhe Institute of Technology (KIT), Germany according to European and German statutory regulations and approved by the Regierungspräsidium Karlsruhe, Germany. BAG-1 exon 1 deleted knockout mice were kindly provided by Michael Sendtner, Institute for Clinical Neurobiology, University of Wuerzburg, Germany (38). BAG-1 exon 2 deleted knockout mice (Bag1tm1a(EUCOMM)Hmgu) were provided by the Infrafrontier European Mouse Mutant Archive (EMMA). Animals were bred using conventional breeding methods, body weight was measured weekly. At the age of three months mice were culled by cardiac puncture. Serum was isolated and testosterone content was analyzed by radioimmunoassay (Bioscientia Healthcare GmbH, Ingelheim, Germany). Subsequently, organs were taken, weighed, and fixed for immunohistochemistry or snap frozen for protein and RNA preparation.

### Microarray analyses (exon 1 BAG-1 knockout mice)

Prostates from BAG-1 exon 1 deleted heterozygous and wild-type control mice castrated for 12 weeks were minced and subjected to total RNA extraction using TRIzol (Invitrogen) and the RNAeasy Mini purification kit (Qiagen). Biological triplicate RNAs were hybridized to a human U133 Plus 2.0 expression array (Affymetrix) at the Dana-Farber Cancer Institute Microarray Core Facility. Gene expression data were normalized and log-scaled using the RMA algorithm and the RefSeq probe definition (39, 40).

### RNA extraction, quantitative reverse transcription PCR (qRT-PCR), and western blotting

RNA extraction, qRT-PCR and western blot were performed as per standard laboratory protocols. Specific details, and primary antibodies and TaqMan probes used, are detailed in Supplementary Table 2 and 3, and Supplementary Materials and Methods.

### Immunohistochemistry

For immunohistochemistry (IHC) studies, androgen receptor full length (AR-FL) and AR splice variant-7 (AR-V7) IHC was performed as previously described (5). Pan-mouse-BAG-1 (panmoBAG-1, mouse, goat polyclonal, AF815, R&D systems), mouse/human AR-FL (AR-FL, mouse/human, rabbit monoclonal, EPR1535 (2), abcam) and pan-BAG-1 (panBAG-1, human, rabbit monoclonal, RM356, RevMAb) were all validated and optimized for IHC in this study. Specific details on the IHC assays developed and quantification are detailed in Supplementary Table 4.

### RNA-sequencing and analysis (BAG-1 knockout mice)

From 1 μg of total RNA we pulled down polyadenylated RNAs with poly-dT magnetic beads. We then prepared sequencing libraries with the TrueSeq Stranded mRNA kit (Illumina) following manufacturer protocol. These libraries were sequenced in paired-end mode (2x50 cycles) with a Hiseq1500 sequencer (Illumina). Raw sequencing data were demultiplexed with Bcl2fastq (version 2.17.1.14, Illumina). Paired end raw reads in FASTQ format were aligned to the reference mouse genome (mm9) using RNA sequencing spliced read mapper TopHat (v2.0.7), with default settings. Differential gene expression and individual gene and transcript expression in units of FPKM (fragments per kilobase of transcript per million mapped reads) were calculated using Cuffdiff (Cufflinks v2.2.1), with default settings. The expressed genes (median expression in either control or treatment samples > 0; n = 17459) were ranked from high to low using the fold change (log_2_), and subsequently used for pathway analysis. Pathway analysis was performed using the GSEA Pre-Ranked algorithm from GSEA software (v4.1.0). GSEA Pre-Ranked results were obtained using the H collection of hallmark gene sets and the C2 collection of curated gene sets (MSigDB v7.1), with default parameters. H and C2 collections were previously mapped to mouse orthologs using the HGNC Comparison of Orthology Prediction tool (https://www.genenames.org/tools/hcop/).

### Compounds

Enzalutamide was purchased from MedChemExpress. Thio-2 (compound A1B1) was synthesized as previously described and provided by N.J (author) (27).

### *In-vivo* Thio-2 toxicity studies

Non tumor bearing NSG male mice were treated with vehicle (5% DMSO in 10% (w/v) HBC (2-hydroxypropyl-β-cyclodextrin) in 0.9% saline) or 15mg/kg Thio-2 by once daily intraperitoneal injection for 5 days with daily weights. Following 5 days treatment mice were sacrificed and organ (heart, kidney, testes, seminal vesicles, and prostate) weights were determined.

### *In-vivo* LNCaP shRNA clone and patient derived xenograft studies

For LNCaP shRNA clone *in-vivo* studies, NSG male mice were inoculated subcutaneously with LNCaP shRNA control or BAG-1 clones and growth was determined between day 16 and 27 once tumors were established. For CP50 patient derived xenografts (PDX), fragments of CP50 tumors were grafted subcutaneously into NSG male mice and drug treatment commenced with vehicle (5% DMSO in 10% (w/v) HBC (2-hydroxypropyl-β-cyclodextrin) in 0.9% saline) or 15 mg/kg Thio-2 by once daily intraperitoneal injection when tumors reached a size of 300 to 400 mm^3^. Mice were treated daily for 5 days (pharmacodynamic analyses) or 14 days (efficacy analyses). After treatment mice were sacrificed, and plasma and tumors were collected for pharmacodynamic analyses.

### *In-vivo* PDX serum PSA analyses

Serum was separated by 5min centrifugation at 9000rpm from blood collected from mice by cardiac puncture under general terminal anesthesia after blood clotting was allowed to take place for 15min. Serum PSA was analyzed in 1:100 diluted serum using the human PSA SimpleStepTM ELISA kit (Abcam) following the manufacturer’s instructions.

### Cell lines

All cell lines used in this study (except for LNCaP95 which were a kind gift from Drs. Alan K Meeker and Jun Luo) were obtained from American Type Culture Collection and grown in recommended media at 37°C in 5% CO_2_ as detailed in Supplementary Table 5. Cell lines were grown from early passages, tested for mycoplasma using the VenorGem One Step PCR Kit (Cambio), and short tandem repeat profiled at regular intervals. LNCaP shRNA clones were developed as previously described, clone C2 (control shRNA) and clone 506 (BAG-1 shRNA) were used for this study (29, 41).

### RNA-sequencing and analysis (cell line)

For LNCaP shRNA clone experiments, LNCaP shRNA clones were grown in full media (10% fetal bovine serum) and three biological replicates used. For unstimulated LNCaP cell experiments, LNCaP cells were grown in full media (10% fetal bovine serum) prior to treatment with vehicle (DMSO 0.1 %) or 50 μM Thio-2 for 17 hours. For stimulated LNCaP cell experiments, LNCaP cells were grown in starved media (10% charcoal stripped serum) for 72 hours prior to treatment with vehicle (DMSO 0.1 %) or 5 μM Thio-2. Following 1 hour pre-treatment with vehicle or 5 μM Thio-2; cells were treated with vehicle (Ethanol 0.1 %) or 10 nM dihydrotestosterone (DHT) for 16 h thereafter (17 hours total treatment). Following treatments, cells were harvested and lysed, and RNA was extracted using Qiagen RNAeasyPlus RNA extraction kit Mini as per manufacturer’s instruction. RNA quality was analyzed using the Agilent Tapestation RNA ScreenTape. 500 ng of total RNA from each sample was first used in the NEBNext rRNA Depletion Kit followed by the NEBNext Ultra II Directional RNA Library Prep Kit, according to the manufacturer’s instructions. Library quality was confirmed using the Agilent Tapestation High Sensitivity DNA ScreenTape. The libraries were quantified and normalized by qPCR using the KAPA Library Quantification Kit (Roche). Library clustering was performed on a cBot with Illumina HiSeq PE Cluster Kit v3. The libraries were sequenced as paired-end 101 base pair reads on an Illumina HiSeq 2500 with an Illumina HiSeq SBS Kit v3. Base calling and quality scoring were performed using Real-Time Analyses (version 1.18.64) and FASTQ file generation and de-multiplexing using CASAVA. Paired end raw reads in FASTQ format were aligned to the reference human genome (GRCh37/hg19) using RNA sequencing spliced read mapper TopHat (v2.1.0), with default settings. The library and mapping quality were assessed using Picard tools (http://broadinstitute.github.io/picard). Differential gene expression was calculated using Cuffdiff (Cufflinks v2.2.1), with default settings. The expressed genes (median gene expression level (FPKM) in either control and Thio-2 treated samples > 0; number of genes = 25635) were ranked from high to low using the fold change (log_2_), and subsequently used for pathway analysis. Pathway analysis was performed using the Gene Set Enrichment Analysis (GSEA) Pre-Ranked algorithm from GSEA software (v4.1.0). GSEA Pre-Ranked results were obtained using the H collection of Hallmark gene sets (MsigDB v7.0), with default parameters.

### Chromatin immunoprecipitation-sequencing and analysis

Briefly, chromatin immunoprecipitation-sequencing (ChIP-seq) libraries were generated using the ThruPLEX DNA-seq kit (Rubicon Genomics) and were sequenced on the Illumina NextSeq 500 platform at the Molecular Biology Core Facility (Dana-Farber Cancer Institute). All samples were processed through the computational pipeline developed at the Dana-Farber Cancer Institute Center for Functional Cancer Epigenetics (CFCE) using primarily open-source programs (42, 43). Sequence tags were aligned with Burrows-Wheeler Aligner (BWA) to build hg19 and uniquely mapped, non-redundant reads were retained. These reads were used to generate binding sites with Model-Based Analysis of ChIP-Seq 2 (MACS v2.1.1.20160309), with a q-value (FDR) threshold of 0.01 (44, 45). A read per million (RPM) normalized BedGraph signal track file generated by MACS2 is further converted to a BigWig file with bedGraphToBigWig (46). Deeptools is used for the plots heatmap (47).

### Small interfering (si) RNA

Cells were transiently transfected with siRNA as indicated. All siRNA were ON-TARGETplus pools (Dharmacon; Horizon), listed in Supplementary Table 6. The siRNA was used along with RNAiMax transfection reagent (ThermoFisher) as per manufacturer’s instructions and incubated with cells as indicated.

### Development of BAG-1 CRISPR knockout 22Rv1 and LNCaP95 cells

22Rv1 and LNCaP95 BAG-1 CRISPR knockout cells were developed following the manufacturers protocol. Briefly, 100000 cells were transfected with BAG-1 sgRNA (6μM; Synthego) and Cas9 2NLS (0.67μM; Synthego) using the 4D Nucleofector System (Lonza Bioscience). After 48 hours, transfection efficiency was assessed in cells transfected with pmaxGFP (0.4μg; Synthego) using fluorescence microscopy, and sgRNA/Cas9 transfected cells were plated in 96-well plates (one cell per well) for clonal expansion. Visual monitoring of single-cell-derived clones was performed daily. The clones that were clearly derived from single cells were screened for BAG-1 protein levels by western blot and selected for study based on BAG-1 protein knockdown efficiency. Details of the BAG-1 sgRNAs used are listed in Supplementary Table 7. Cells transfected with Cas9 2NLS complexed with no sgRNA were used as control.

### *In-vitro* cell line proliferation

Cell proliferation was measured in parental, BAG-1 CRISPR knockout or siRNA treated prostate cancer cell lines in response to vehicle (0.1% DMSO) and Thio-2 (at various concentrations) using CellTiter-Glo (Promega) according to the manufacturer’s instructions. Briefly, cells were plated in 96-well plates. For siRNA treated cells, 24 hours after siRNA transfection, cells were seeded and subsequently (24 hours later) treated with either vehicle or Thio-2 in medium. For CRISPR clones and parental prostate cancer cell lines, siRNA transfection was omitted. CellTiter-Glo® Luminescent Cell Viability Assay (Promega) or CyQUANT™ Cell Proliferation Assay (ThermoFisher) were used to assay growth according to the manufacturer’s instruction on day 0 or after 4 or 6 days of treatment and luminescence was measured using Synergy HTX (BioTek).

### Androgen receptor N-terminus and Thio-2 binding

NMR spectra were recorded at 278 K on either a Bruker 800 MHz Avance NEO or a 600 MHz Bruker Avance III spectrometer, equipped with TCl cryoprobes. Intensities and chemical shift perturbations (CSP) were obtained from ^1^H,^15^N correlation experiments and calculated using the following equation:

$$CSP=\sqrt{{\left(\delta H\right)}^{2}-{\left(\frac{\delta N}{5}\right)}^{2}}$$

All spectra were referenced using DSS. NMR spectra were obtained for 25 μM AR NTD constructs NTD_1-518_ and NTD_330-447_ (Tau-5*) in the present and absence of 250 μM Thio2 and EPI-001. Samples were prepared in phosphate buffer (20 mM sodium phosphate, pH 7.4, 1 mM TCEP, 0.05 % NaN_3_), containing 10 % D_2_O, 10 μM DSS and 0.5 % DMSO-d_6_. Experiments with ^15^N-labelled AR NTD constructs NTD_1-518_ and NTD_330-447_ at 25 μM were mixed with a 10 molar excess equivalents (250 μM) of Thio-2 or EPI-001 (positive control) and measured at 5°C.

### Plasmids

The plasmids used in this study were the pReceiver-M13 vector carrying a C-terminal fusion FLAG-tag (GeneCopoeia) containing either an empty cassette as control (CONTROL-FLAG) or the AR-FL (AR-FL-FLAG). An AR-V7 C-terminal fusion FLAG-tag (AR-V7-FLAG) was generated from the corresponding AR-FL-FLAG plasmid through restriction enzyme digest (XhoI, BSTE1I; New England Biosciences) and ligation techniques. The resulting plasmids were verified by sequencing (Beckman Coulter Genomics). The ARE3-PSA-luciferase (PSA-Luc) reporter plasmid has been previously described (48).

### PSA luciferase reporter assay

PC3 cells were seeded at a density of 5000 cells per well in 96-well plates in phenol-red free RPMI media supplemented with 10% charcoal stripped fetal bovine serum and concurrently transfected with either 0.5 μg/ml CONTROL-FLAG, AR-FL-FLAG or AR-V7-FLAG, and 0.25 μg/ml PSA-luc, using X-tremeGENE HP DNA Transfection Reagent (Merck) as per manufacturers recommendations and incubated overnight. Cells were then treated with either vehicle (DMSO 0.1 %), various concentrations (5, 10 or 50 μM) of Thio-2 or 5 μM enzalutamide for 1 hour prior to stimulation with or without 10nM dihydrotestosterone (DHT). Cells were then incubated for 16 hours and then lysed with Pierce IP Lysis Buffer (ThermoFisher Scientific). Luciferase Assay Reagent (Promega) was added to lysate and luciferase activity was measured using the BioTek Cytation 5 Cell Imaging Multimode Reader (Agilent).

### Thio-2 solubility

Thio-2 solubility was measured by comparing Thio-2 aromatic signals (region 6.5-8 ppm) to DSS signal (internal reference, at 0 ppm) in 1D ^1^H spectra. Samples containing variable concentrations of Thio-2 were prepared in NMR buffer, containing 10 % D_2_O, 10 μM DSS and 0.5 or 2 % DMSO-d_6._ Samples were measured on 600 MHz spectrometer at 278, 298 and 310 K. Integration of Thio-2 ^1^H aromatic signals (region 6.5-8 ppm) and the internal reference (DSS) ^1^H signal (at 0 ppm) were used for quantification. Samples containing 5 μM Thio-2, 10 μM DSS, and variable amounts of DMSO-d_6_ (buffer 20 mM sodium phosphate (pH 7.4), 1mM TCEP, 10% D_2_O, 0.05% NaN_3_) were recorded on 600 MHz Bruker Avance spectrometer equipped with a cryoprobe.

### Statistical analyses

Kruskal-Wallis test was used to determine the difference between ligandable properties of BAG-1, BCL2 and druggable protein kinase ATP site. Unpaired Student t-tests were used to determine the difference between mRNA expression of BAG-1 exon 2 deleted knockout and wildtype mice. Overall survival of BAG-1 exon 2 deleted knockout and wildtype mice were estimated using the Kaplan–Meier method, and respective hazard ratios were obtained by Cox regression. Unpaired Student t-tests were used to determine differences between characteristics of BAG-1 exon 2 deleted knockout and wildtype mice. Unpaired Student t-tests were used to determine differences between DHT stimulated genes in LNCaP shRNA clones. Unpaired Student t-tests were used to determine differences in growth between LNCaP shRNA clones *in-vitro* at day 27. Unpaired Student t-tests were used to determine the difference between growth of PDX organoids (PDX-Os), PDXs and prostate cancer cell lines (with siRNA control/BAG-1 and CRISPR control/BAG-1) treated with vehicle or Thio-2. Unpaired Student t-tests were used to determine the difference between mRNA expression of PDX-Os and prostate cancer cell lines (with siRNA control/BAG-1 and CRISPR control/BAG-1) treated with vehicle or Thio-2. Unpaired Student t-tests were used to determine the difference between weights of non-tumor bearing mice and organs treated with vehicle or Thio-2. The doubling time (2-fold growth) for CP50 PDXs were used as a surrogate endpoint for overall survival. Overall survival was estimated using the Kaplan–Meier method, and respective hazard ratios were obtained by Cox regression. Unpaired Student t-tests were used to determine the difference between serum PSA of PDXs treated with and without Thio-2. Bioinformatic analyses are detailed in associated sections. Statistical analyses were performed with GraphPad Prism Version 7 (GraphPad Software). All experimental replicates and statistical analyses performed are detailed in figure legends. Statistical significance was pre-specified at P ≤ 0.05. No adjustment for multiple testing has been made.

### Study approvals

All patients treated at The Royal Marsden Hospital had provided written informed consent and were enrolled in institutional protocols approved by the Royal Marsden NHS Foundation Trust Hospital (London, United Kingdom) ethics review committee (reference 04/Q0801/60). All mouse work was carried out in accordance with the The Institute of Cancer Research (ICR) guidelines, including approval by the ICR Animal Welfare and Ethical Review Body, and with the UK Animals (Scientific Procedures) Act 1986, and/or in accordance with the German national and KIT institutional guidelines, including approval by the KIT Animal Welfare and Ethical Review Body, and the Regierungspräsidium Karlsruhe, Germany.

## Results

### BAG-1 is highly expressed and associates with signaling pathways critical for the development and progression of castration resistant prostate cancer

**Figure 1:**
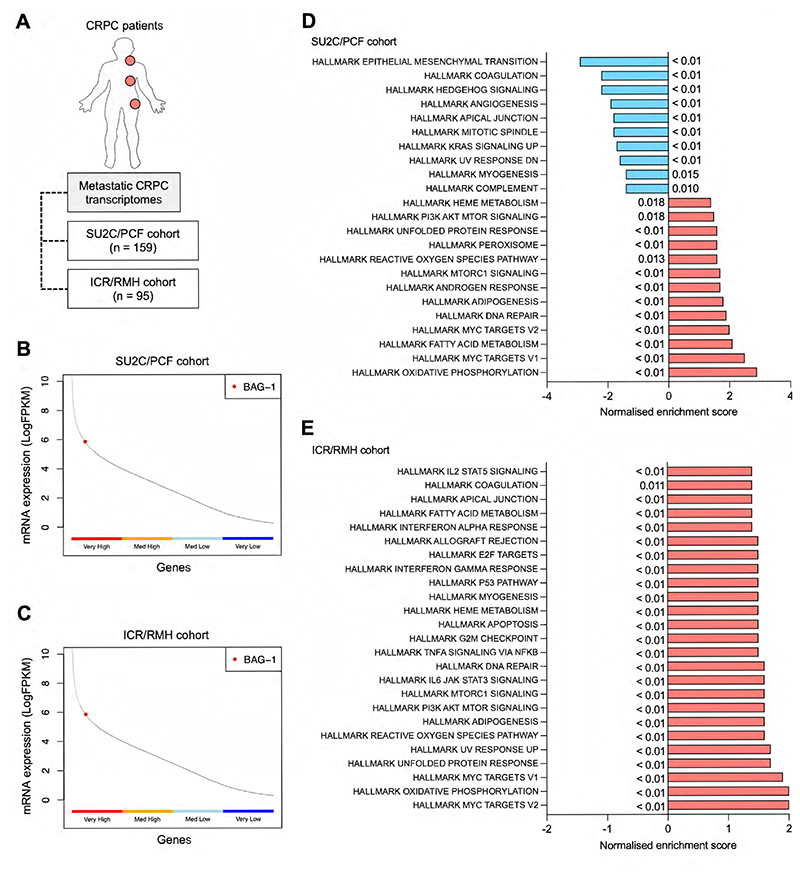
BAG-1 is highly expressed and associates with signaling pathways critical for the development and progression of castration resistant prostate cancer. **(A)** Two independent castration resistant prostate cancer (CRPC) patient transcriptome cohorts were used in this study. The Stand Up To Cancer/Prostate Cancer Foundation (SU2C/PCF) patient cohort included RNA-sequencing (RNA-seq) on 159 CRPC biopsies and the Institute of Cancer Research/Royal Marsden Hospital (ICR/RMH) patient cohort included RNA-seq on 95 CRPC biopsies. **(B-C)** SU2C/PCF (B) and ICR/RMH (C) CRPC transcriptome analyses for BAG-1 mRNA expression compared to the 20,000 highest expressed genes divided into very high (upper 25% expressed genes), medium high (50%−75% expressed genes), medium low (25%−50% expressed genes) and very low (lower 25% expressed genes). **(D-E)** Gene Set Enrichment Analysis (GESA) shows BAG-1 mRNA levels association with hallmark pathways in SU2C/PCF (D) and ICR/RMH (E) cohorts. Normalized enrichment scores and false discovery rates (FDR) are shown. Hallmark pathways significantly (FDR ≤ 0.05) enriched and de-enriched with BAG-1 mRNA expression are shown.

To investigate the importance of BAG-1 isoforms in castration resistant prostate cancer (CRPC), we interrogated the association between BAG-1 mRNA expression and the Molecular Signatures Database hallmark gene collection in two independent CRPC patient transcriptome cohorts (Figure 1A) (30–33). BAG-1 mRNA was highly expressed (top 25% expressed genes) in both CRPC transcriptome cohorts (Figure 1B-C). Furthermore, BAG-1 mRNA expression positively correlated with multiple signaling pathways implicated in the development and progression of CRPC; including MYC targets V1 and V2, E2F targets, IL6 JAK STAT signaling, DNA repair, PI3K AKT MTOR signaling, MTORC1 signaling and androgen response (Figure 1D-E) (30–32). Taken together, these data demonstrate that BAG-1 isoforms are highly expressed and associate with signaling pathways implicated in the development and progression of CRPC.

### The BAG domain of the BAG-1 isoforms present a groove that provides a tractable but challenging drug target

**Figure 2:**
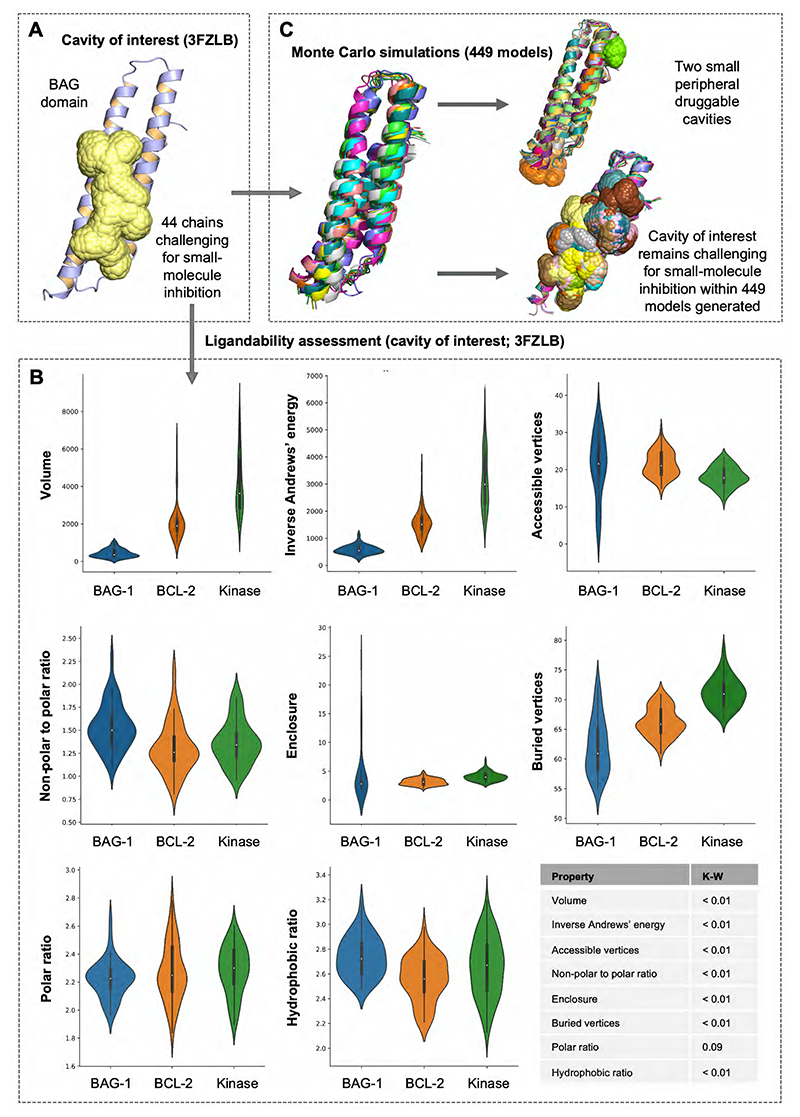
Druggability assessment of the BAG domain of BAG-1. **(A)** Visualization of the BAG domain cavity of interest identified by canSAR using the 44 3-dimensional (3D) HSC70-BAG domain structures available mapped onto the representative structure (PDB ID 3FZLB). The cavity of interest as volume surface (in yellow) is shown on the BAG domain (violet) of BAG-1. **(B)** Key geometric and physicochemical parameters for the cavity of interest within the BAG domain (blue), a druggable protein-protein interaction (BCL-2; orange) and the druggable kinase ATP site (green) are shown as violin plots. P values were calculated using the Kruskal-Wallis (K-W) test. **(C)** Monte Carlo simulations identified 449 models with 4489 cavities for the original 44 3D HSC70-BAG domain structures.

Having demonstrated that BAG-1 isoform expression associates with critical signaling pathways in CRPC, we expanded our previous studies to investigate whether BAG-1 represents a tractable drug target (15). Therapies targeting the highly conserved BAG domain, present in all BAG-1 isoforms and critical for BAG-1 isoform function, provides an attractive strategy to abrogate BAG-1 activity in CRPC (8–10, 15–22). We had previously reported that the BAG domain of BAG-1 isoforms presents a groove, suitable for peptide or peptido-mimetic modulators, but maybe challenging for small molecule inhibition (15). Updated canSAR analysis demonstrate that the 44 3-dimensional (3D) HSC70-BAG domain structures continue to reveal a lack of a classical ‘ligandable’ cavity within the BAG domain (Figure 2A, Supplementary Table 1) (15, 34, 35, 49, 50). We find that the properties (except polar ratio) of the BAG domain groove fall outside the distributions expected for druggable cavities (all P < 0.01, Kruskal-Wallis test) (Figure 2B) (15, 36, 51). We next wanted to explore whether a cryptic druggable cavity might emerge should we probe the structural fluctuations of the protein. To this end, we performed Monte Carlo simulations, which identified 4489 cavities in 449 generated models (37). Despite allowances for structural fluctuations, the cavity of interest (80% of amino acid residues within the original pocket; 3FZLB) remains challenging for small molecule inhibition (Figure 2C). Furthermore, of the remaining cavities identified, only seven (of 4489) cavities have been identified as ligandable, and these may represent artifacts as they are only identified in a limited number of models (six of 449) derived (Figure 2C). These updated analyses confirm that the BAG domain provides a tractable although challenging drug target with geometric and physicochemical properties that may require peptide or peptido-mimetic approaches.

### BAG-1 isoform knockout impacts mouse hormone physiology suggesting that targeting the BAG domain should be associated with minimal ‘on-target’ toxicity

**Figure 3:**
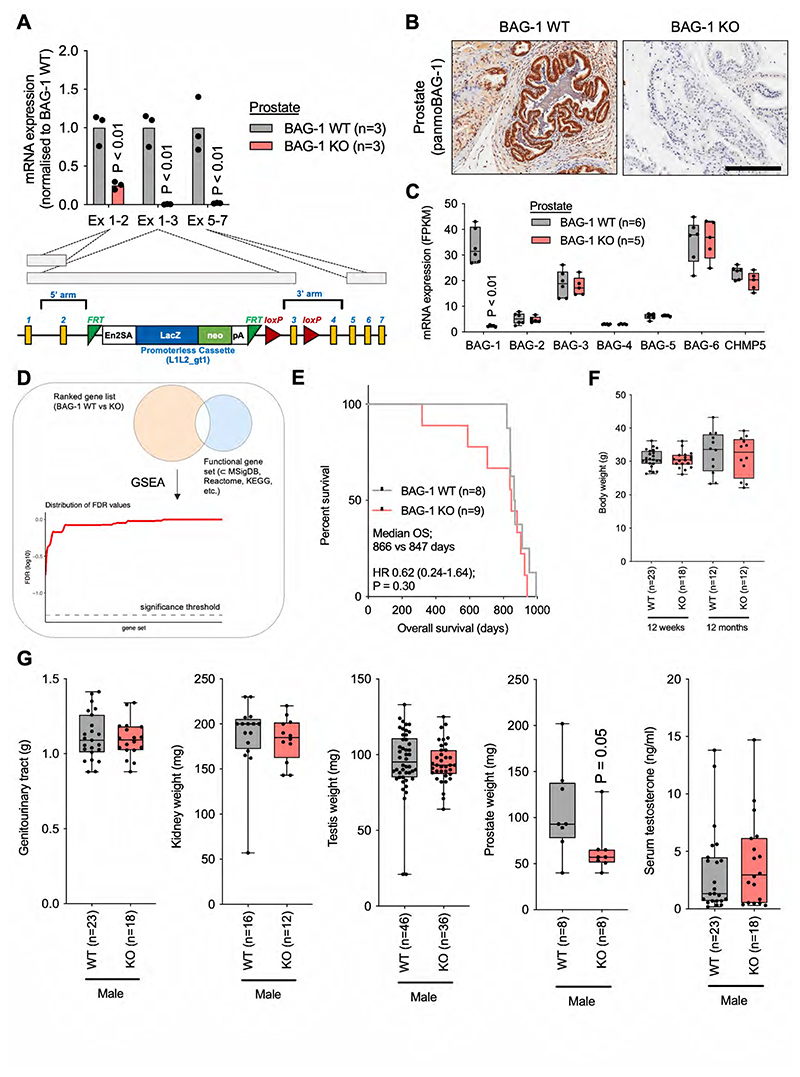
BAG-1 knockout male mice are fertile and viable with reduced prostate weight. **(A)** BAG-1 knockout KO (BAG-1 KO) mouse strain Bag1tm1a(EUCOMM)Hmgu was developed by the European conditional mouse mutagenesis (EUCOMM) program by insertion of an artificial exon containing the coding sequence of beta-Geo, a fusion protein of beta-Galactosidase (LacZ) and neomycin (neo) followed by a stop codon and polyadenylation (pA) sequence between exon 2 and exon 3 flanked by Flp recognition sites disrupts the expression of the WT gene, replacing the endogenous BAG-1 expression by a fusion protein of the BAG-1 N-terminal sequence encoded in exon 1 and 2 and beta-Geo. Mouse prostates from BAG-1 knockout KO (BAG-1 KO) and BAG-1 wildtype (BAG-1 WT) male mice were analyzed for BAG-1 mRNA (quantitative reverse transcriptase-polymerase chain reaction; qRT-PCR) levels. BAG-1 exon 1 to 2 (Ex 1-2), exon 1 to 3 (Ex 1-3) and exon 5 to 7 (Ex 5-7) mRNA was quantified for BAG-1 KO (red bars; n=3) and BAG-1 WT (gray bars; n=3) mice. mRNA expression was calculated relative to mouse GAPDH and normalized to BAG-1 WT. Mean levels from three prostates are shown. P values were calculated for BAG-1 KO compared with BAG-1 WT mice using unpaired Student t-test. P values ≤ 0.05 are shown. **(B)** Prostates from BAG-1 knockout (BAG-1 KO) mouse strain Bag1tm1a(EUCOMM)Hmgu and BAG-1 WT male mice were analyzed for mouse (mo) BAG-1 protein (immunohistochemistry) levels. Representative micrographs of BAG-1 detection in mouse prostates by pan-mouse-BAG-1 (panmoBAG-1) antibody IHC are shown. Scale bar, 200 μm. **(C)** Mouse prostates from BAG-1 KO (red bars; n = 5) and BAG-1 WT (gray bars; n = 6) male mice at age 12 weeks were taken and samples prepared for RNA sequencing. Median BAG isoforms and CHMP5 mRNA levels (fragments per kilobase of transcript per million reads; FPKM) with interquartile range, and smallest and largest value, is shown. P values were calculated for BAG-1 KO compared with BAG-1 WT mice using unpaired Student t-test. P values ≤ 0.05 are shown. **(D)** Overview of gene set enrichment analysis (GSEA) using MsigDB (v7.0) functional pathways: H, Hallmark; C2, Curated Gene Sets (including KEGG, Biocarta, Reactome). BAG-1 KO and BAG-1 WT RNA-sequencing (RNA-seq) analysis was compared, change in gene expression was ranked by log_2_ fold change, then tested for enrichment against functional gene sets (H, C2), using GSEA. The distribution of resulting FDR values (log_10_) is shown in red, and the threshold for significance (FDR 0.05) is shown by dotted grey line. None of the pathways tested reached the significance threshold. **(E)** Kaplan-Meier curves of overall survival (OS) of BAG-1 KO (red line; n=9) and BAG-1 WT (gray line; n=8) male mice from birth. Median OS, hazard ratio (HR) with 95% confidence intervals and P values for univariate Cox survival model are shown. **(F)** The body weight of male BAG-1 KO (red bars) and BAG-1 WT (gray bars) male mice at 12 weeks and 12 months was determined. Median body weight with interquartile range, and smallest and largest value, is shown. P values were calculated for BAG-1 KO compared with BAG-1 WT mice using unpaired Student t-test. P values ≤ 0.05 are shown. **(G)** The weight of the genitourinary tract, kidney, testis, prostate, and serum levels of testosterone, from male BAG-1 KO (red bar) and BAG-1 WT (gray bar) male mice at age 3 months and older was determined. Median weight or serum testosterone levels with interquartile range, and smallest and largest value, is shown. P values were calculated for BAG-1 KO compared with BAG-1 WT mice using unpaired Student t-test. P values ≤ 0.05 are shown.

BAG-1 is a multifunctional protein that binds numerous molecular targets to regulate a plethora of cellular processes (8–10). In light of this, one critical consideration is that therapies blocking BAG-1 function may be associated with modulation of normal physiology resulting in treatment related adverse events. To further investigate this, we studied the impact of BAG-1 loss in BAG-1-deleted mouse models. Targeted deletion of exon 1 and 2 of the BAG-1 gene has previously been reported, with BAG-1 homozygous deletion reported to be embryonically lethal (38). Analyzing these generated and previously reported BAG-1 heterozygous mice that are viable, we demonstrate that BAG-1 mRNA is indeed reduced but surprisingly CHMP5 (which is located on the opposite strand of chromosome 9 to BAG-1) is also downregulated in this model (Supplementary Figure 1A) (10, 52). CHMP5 deletion has been previously shown to be embryonically lethal and therefore may explain the phenotype previously reported for this BAG-1 knockout model (52). Considering this, we explored an alternative knock-out strategy. We utilized a BAG-1 specific knockout-first mouse strain Bag1tm1a(EUCOMM)Hmgu (referred to as BAG-1 knockout from here on out), developed by the European Conditional Mouse Mutagenesis (EUCOMM) Program to study the impact of losing just BAG-1 (Figure 3A). These BAG-1 knockout (KO) mice are viable and fertile, with BAG-1 deletion confirmed at the mRNA level, and following pan-mouse-BAG-1 antibody validation, at the protein level including in prostates isolated from knockout mice and littermate controls (Figure 3A-B, Supplementary Figure 1B-C). In contrast to BAG-1 mRNA and protein levels, there was no change in protein expression or localization of the androgen receptor (AR) in prostates from BAG-1 KO mice when compared to WT mice (Supplementary Figure 1C-E). In order to investigate the broader impact of BAG-1 deletion on gene expression and signaling pathways, RNA sequencing was performed on BAG-1 KO and wild type (WT) mouse prostates; this demonstrated significant (P < 0.01, Student t-test) reductions in BAG-1 mRNA with no significant change in CHMP5 or other BAG family members (Figure 3C). There was no significant enrichment in functional pathways in BAG-1 KO compared to WT mouse prostates (Figure 3D). Consistent with this, BAG-1 deletion did not impact mouse overall survival (Figure 3E, Supplementary Figure 2A). The only characteristics that were different following BAG-1 deletion were decreased prostatic weight (P = 0.05, Student t-test), increased duration of pregnancy (P = 0.01, Student t-test), decreased litter size (P = 0.04, Student t-test), increased day 1 neonatal weight (P = 0.02, Student t-test), and decreased neonate survival rate on day 2 (P = 0.04, Student t-test), potentially indicating a role for BAG-1 in hormone physiology phenotypically, although there was no obvious impact on AR levels or localization, and other signaling pathways, when interrogating mouse prostates specifically (Figure 3D and 3F-G, Supplementary Figure 1C-E and 2B-D). In addition, histological analysis of all major organs demonstrated no difference between BAG-1 KO and WT mice (Supplementary Figure 3 and 4). These data suggest that therapies blocking the BAG-1 isoforms may impact hormone physiology phenotypically, which although it needs to be considered, should be associated with limited toxicity.

### BAG-1 isoform knockdown induces a limited phenotype in the LNCaP cell line prostate cancer model

**Figure 4:**
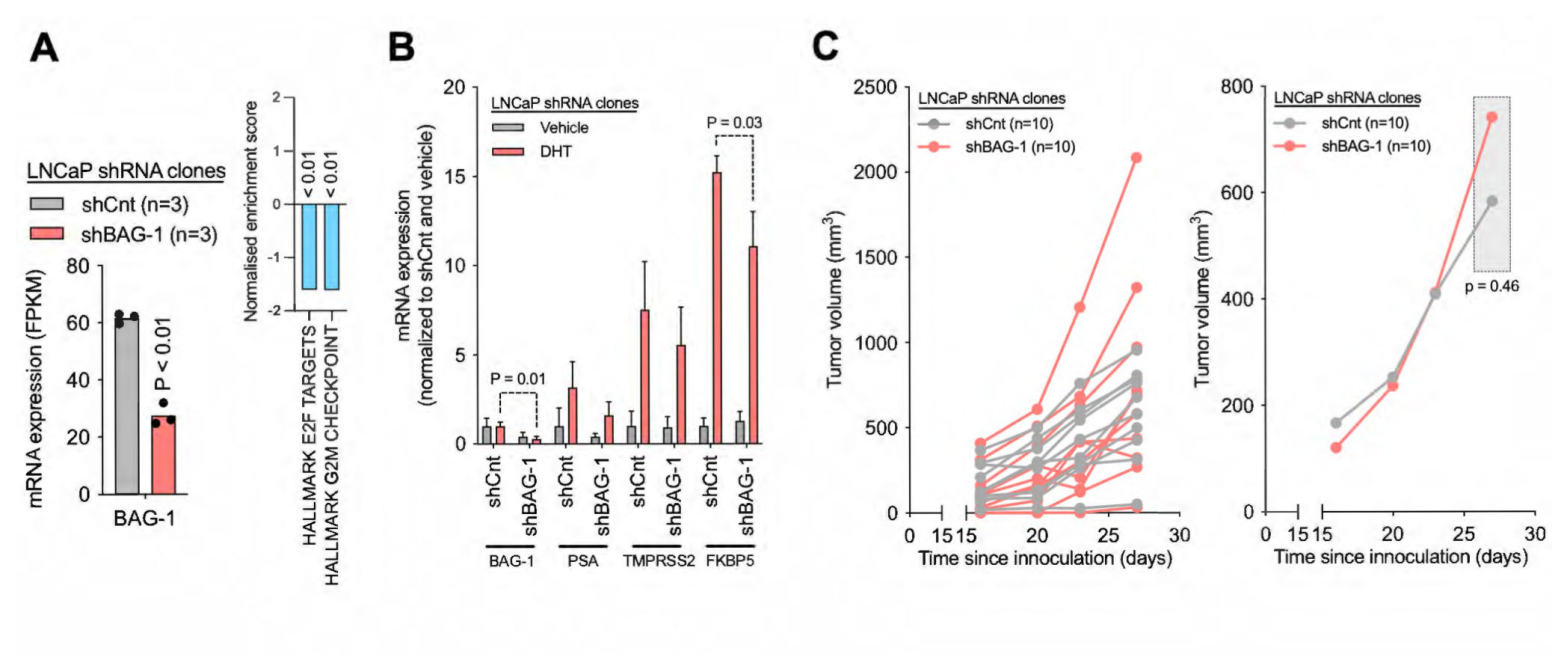
BAG-1 isoform knockdown induces a limited phenotype in the LNCaP cell line prostate cancer model. **(A)** LNCaP shRNA clones were collected and prepared for RNA-seq. Mean BAG-1 mRNA levels (FPKM) in control (gray bars; n = 3) and BAG-1 (red bars; n = 3) shRNA clones is shown. P values were calculated for BAG-1 shRNA (shBAG-1) clones compared to control shRNA (shCnt) clones using unpaired Student t-test. P values ≤ 0.05 are shown. Analysis of RNA-seq with Gene Set Enrichment Analysis (GESA) shows BAG-1 knockdown associates with hallmark pathways. Normalized Enrichment Scores and False Discovery Rates are shown. Hallmark pathways significantly (FDR ≤ 0.05) enriched and de-enriched with BAG-1 knockdown are shown. **(B)** LNCaP shRNA clones were grown in starved media (10% charcoal stripped serum) for 72 hours prior to treatment with vehicle (gray bars; Ethanol 0.1 %) or 10 nM dihydrotestosterone (red bars; DHT) for 16 hours and BAG-1, PSA, TMPRSS2 and FKBP5 mRNA expression was determined. Mean mRNA expression (normalized to average of GAPDH/B2M/HRPT1/RPLP0 and shCnt/vehicle; defined as 1) with standard deviation from three individual experiments is shown. P values were calculated for the impact of BAG-1 knockdown on DHT stimulation using unpaired Student t-test. P values ≤ 0.05 are shown. **(C)** NSG male mice were inoculated with LNCaP shRNA control (shCnt; gray line; n=10) or BAG-1 (shBAG-1; red line; n = 10) clones and growth was measured between day 16 and 27. Growth of all tumors (left) and mean growth (right) is shown. P values were calculated comparing shCnt and shBAG-1 at day 27 using unpaired Student t-test.

Considering the marked differences between BAG-1 associated signaling pathways in our patient correlative data and mouse knockout studies, we explored the impact of BAG-1 knockdown in LNCaP shRNA clones (Figure 4A). RNA sequencing was performed on LNCaP control and BAG-1 shRNA clones; this demonstrated a significant (P < 0.01, Student t-test) reduction in BAG-1 mRNA expression (Figure 4A). There was de-enrichment in E2F targets and G2M checkpoint signatures that were both positively correlated with BAG-1 mRNA expression in our Institute of Cancer Research/Royal Marsden Hospital (ICR/RMH) CRPC transcriptome cohort (Figure 1E and 4A). However, there was no significant alternations in other functional pathways associated with BAG-1 mRNA expression in our patient CRPC transcriptome studies when comparing BAG-1 knockdown and control LNCaP shRNA clones (Figure 1D-E and 4A). Next, we explored the impact of BAG-1 isoform knockdown on specific AR regulated genes (PSA, TMPRSS2 and FKBP5 (Figure 4B). BAG-1 knockdown significantly reduced dihydrotestosterone (DHT) mediated induction of FKBP5 (P = 0.03, Student t-test) but had no significant impact on PSA and TMPRSS2 (Figure 4B). Finally, we interrogated the impact of BAG-1 isoform knockdown on the growth of LNCaP cells *in-vivo*, demonstrating there was no significant difference in the growth of BAG-1 knockdown and control LNCaP shRNA clones (Figure 4C). These data suggest that BAG-1 isoform knockdown induces a limited phenotype in this specific context and that targeting BAG-1 as a therapeutic target for lethal prostate cancer requires further interrogation.

### Thio-2 inhibits the growth of castration resistant prostate cancer patient derived models with associated suppression of androgen receptor target genes

**Figure 5:**
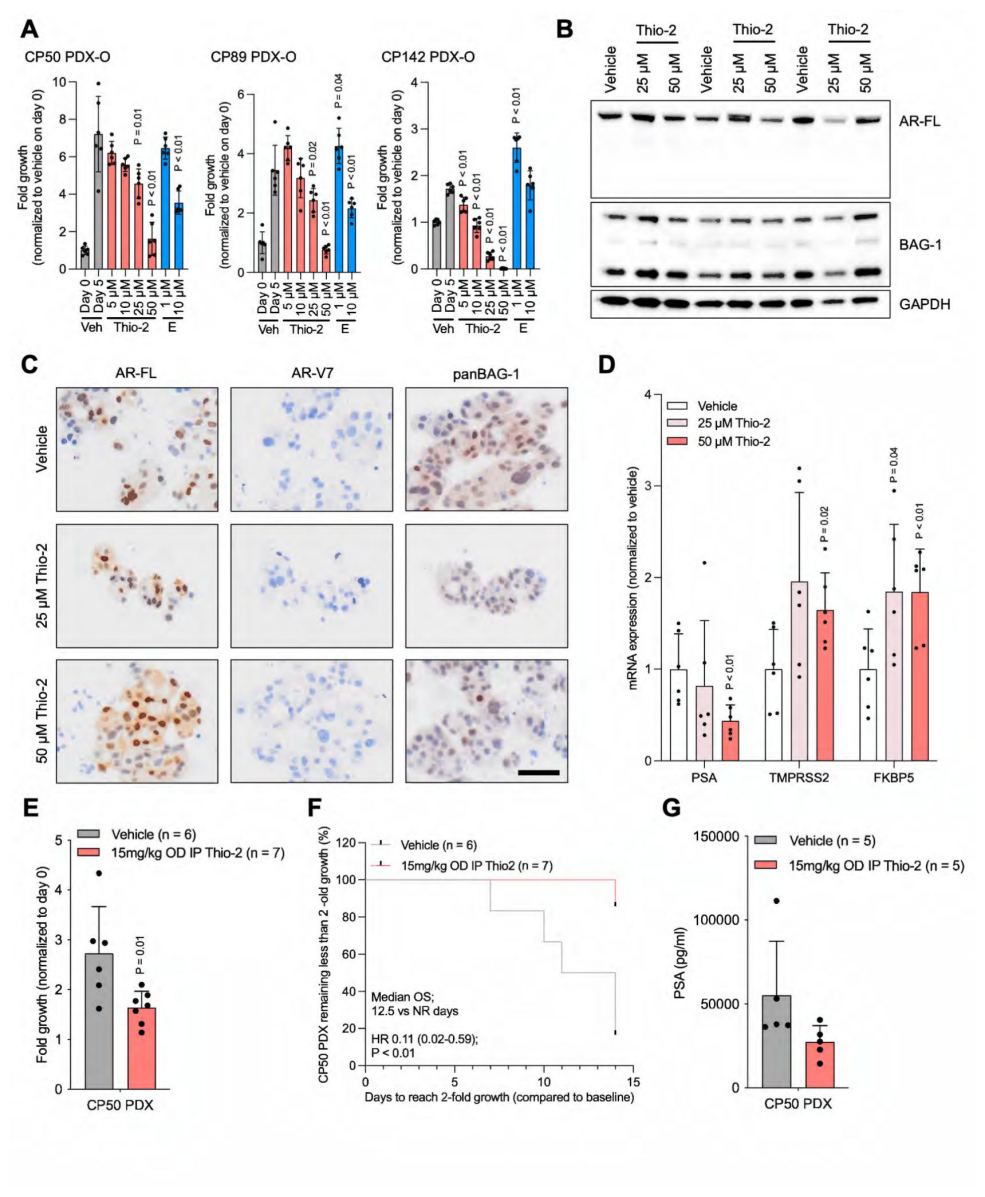
Thio-2 inhibits the growth of castration resistant prostate cancer patient derived models with associated suppression of androgen receptor target genes. **(A)** CP50, CP89 and CP142 patient derived xenograft organoids (PDX-Os) were treated with vehicle (Veh, DMSO 0.1 %), various concentrations (5, 10, 25 and 50 μM) of Thio-2 or various concentrations (1 and 10 μM) of enzalutamide (E), and growth determined after 5 days by CellTiter-Glo® 3D Cell Viability Assay. Mean fold change in growth (compared to day 0) with standard deviation from a single experiment with six replicates is shown. P values were calculated for each condition compared to vehicle at 5 days using unpaired Student t-test. P values ≤ 0.05 are shown. **(B)** CP50 PDX-O were treated with vehicle (DMSO 0.1 %) or various concentrations (25 and 50 μM) of Thio-2 for 17 hours. The effect of each condition on AR-FL, BAG-1 and GAPDH protein expression was determined. Single western blot with triplicates is shown. **(C)** Representative micrographs of AR-FL, AR-V7 and pan BAG-1 (panBAG1) detection by immunohistochemistry of formalin-fixed paraffin-embedded CP50 PDX-O treated with vehicle (DMSO 0.1 %) or various concentrations (25 and 50 μM) of Thio-2 for 17 hours are shown. Scale bar: 50 μm. **(D)** CP50 PDX-O were treated with vehicle (DMSO 0.1%) or various concentrations (25 and 50 μM) of Thio-2 for 17 hours. The effect of each condition on PSA, TMPRSS2 and FKBP5 mRNA expression was determined. Mean mRNA expression (normalized to average of GAPDH/B2M/HRPT1/RPLP0 and vehicle treatment; defined as 1) with standard deviation from a single experiment with six replicates is shown. P values were calculated for each condition compared to vehicle using unpaired Student t-test. P values ≤ 0.05 are shown. **(E)** CP50 PDXs were treated with 15mg/kg Thio-2 (n = 7) or vehicle (n = 6) OD IP for 14 days. Mean growth (normalized to day 0; defined as 1) with standard deviation was determined on day 14. P values were calculated comparing 15 mg/kg Thio-2 IP OD treatment arm to vehicle control using unpaired Student t-test. P values ≤ 0.05 are shown. **(F)** The doubling time (2-fold growth) for CP50 PDXs were used as a surrogate endpoint for overall survival (OS). Median OS, Hazard ratio (HR) with 95% confidence intervals and P values for univariate cox survival model are shown. **(G)** The effect of 15 mg/kg Thio-2 OD IP compared to vehicle on serum PSA was determined at 5 days. Mean serum PSA with standard deviation was determined for each mouse. P values were calculated for vehicle compared to 15 mg/kg Thio-2 OD IP using unpaired Student t-test. P values ≤ 0.05 are shown.

Despite the challenging geometric and physicochemical properties associated with targeting the BAG domain of BAG-1 isoforms and limited phenotype associated with BAG-1 isoform knockdown across a number of models, Thio-2 has been postulated to bind the BAG domain and block BAG-1 isoform function, including BAG-1L mediated AR transactivation (15, 26, 53). We therefore explored the impact of Thio-2 on AR signaling and on the growth of patient derived models of CRPC. We utilized three patient derived xenograft (PDX) models, CP50, CP89 and CP142, all of which were developed from lymph node biopsies of patients with CRPC (Supplementary Figure 5A) (54–56). PDX-organoids (PDX-O) were derived from these individual PDX models to support interrogation of Thio-2 *in-vitro*. Having validated a pan-BAG-1 (panBAG-1) antibody for immunohistochemistry (IHC), we demonstrated AR full-length (AR-FL), AR splice variant-7 (AR-V7) and BAG-1 isoform expression across all these PDXs and their related PDX-O models (Supplementary Figure 5B-C). Thio-2 inhibited the growth of PDX-Os from CP50, CP89 and CP142 (Figure 5A). Interestingly, enzalutamide maintained some growth inhibitory effects in CP50 and CP89, but not in CP142, (Figure 5A). In the CP50 PDX-O model, Thio-2 appeared to have little impact on AR-FL and BAG-1 isoform protein expression although it suppressed PSA mRNA levels (at 50 μM) (Figure 5B-D). In addition, AR-V7 protein was not detected in the CP50 PDX-O model *in-vitro* (Figure 5B-C). We next investigated Thio-2 *in-vivo*, first exploring Thio-2 tolerability in non tumor-bearing mice; we administered 15 mg/kg once daily (OD) intraperitoneally (IP) which significantly impacted heart weight (P < 0.01, Student t-test), and reduced other parameters including kidney, testes, seminal vesicles, prostate and body weight, although not significantly (Supplementary Figure 6A-C). We next explored the impact of 15 mg/kg OD IP Thio-2 on AR signaling and growth in the tumor bearing CP50 PDX, to determine if any therapeutic impact was observed (Supplementary Figure 6D). Thio-2 significantly (P = 0.01, Student t-test) decreased tumor growth, and time to reach 2-fold tumor growth, of CP50 PDX compared to vehicle (Figure 5E-F). In addition, Thio-2 treatment also reduced serum and tumor PSA protein levels (Figure 5G, Supplementary Figure 6E). Taken together, these *in-vitro* and *in-vivo* data demonstrate Thio-2 anti-tumor activity and pharmacodynamic modulation of AR signaling in patient derived models of CRPC.

### Thio-2 downregulates critical pathways, including androgen receptor signaling, implicated in prostate cancer development and progression

**Figure 6:**
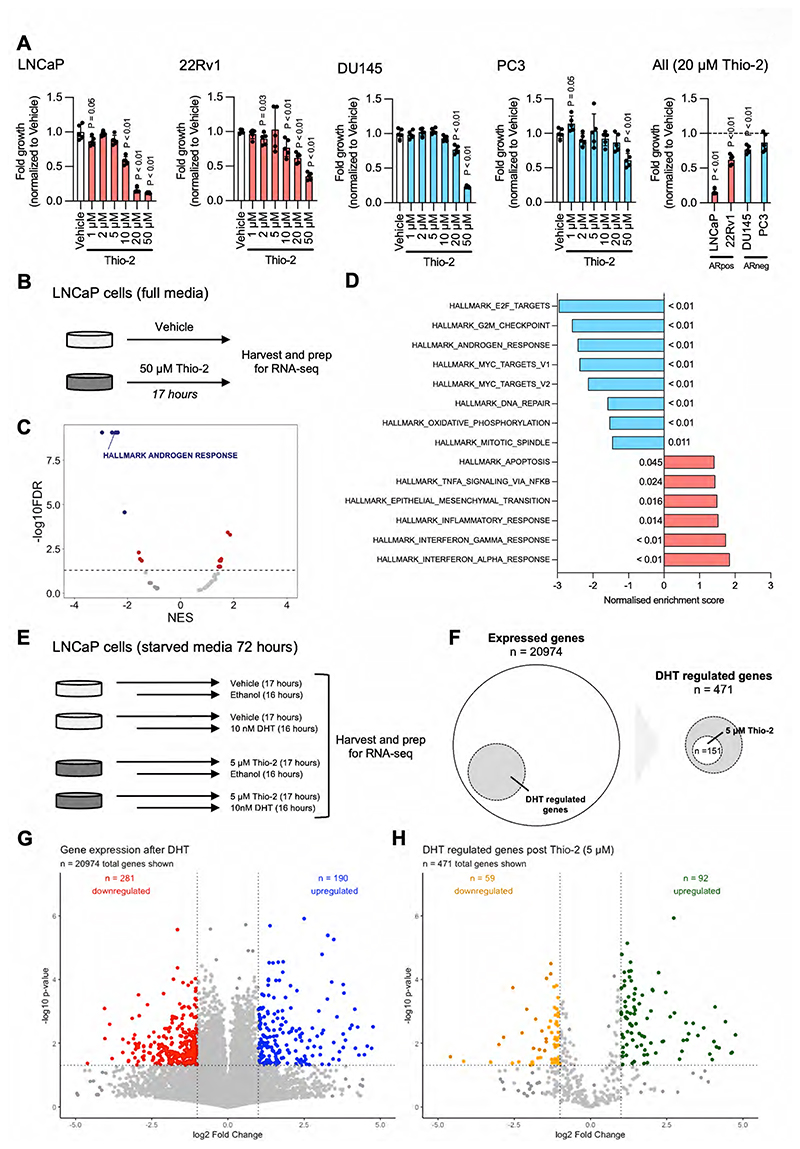
Thio-2 downregulates critical pathways, including androgen receptor signaling, implicated in castration resistant prostate cancer development and progression. **(A)** Androgen receptor positive (ARpos; LNCaP and 22Rv1) and AR negative (ARneg; DU145 and PC3) prostate cancer cells were treated with vehicle (DMSO 0.1 %) or various concentrations (1, 2, 5, 10, 20 and 50 μM) of Thio-2 and growth was determined after 4 days by CyQUANT™ Cell Proliferation Assay. Mean fold change in growth (compared to vehicle) with standard deviation from a single experiment with five replicates is shown. P values were calculated for each condition compared to vehicle unpaired Student t-test. P values ≤ 0.05 are shown. **(B)** Schematic of RNA-sequencing (RNA-seq) experimental setup. LNCaP cells were grown in full media (10% fetal bovine serum) prior to treatment with vehicle (DMSO 0.1 %) or 50 μM Thio-2 for 17 hours. RNA-sequencing was performed on a single experiment in triplicate. **(C)** Analysis of RNA-seq with Gene Set Enrichment Analysis (GESA) shows Thio-2 treatment associates with hallmark pathways. Normalized Enrichment Scores (NES) and False Discovery Rates (FDR) are shown. Dotted line indicates significant threshold (FDR 0.05). Colored dots denote significant Hallmark pathways enriched and de-enriched with Thio-2 treatment. **(D)** Hallmark pathways significantly (FDR ≤ 0.05) enriched and de-enriched with Thio-2 treatment are shown. **(E)** Schematic of RNA-seq experimental setup. LNCaP cells were grown in starved media (10% charcoal stripped serum) for 72 hours prior to treatment with vehicle (DMSO 0.1 %) or 5 μM Thio-2. Following 1 hour pre-treatment with vehicle or 5 μM Thio-2; cells were treated with vehicle (Ethanol 0.1 %) or 10 nM dihydrotestosterone (DHT) for 16 hours (17 hours total treatment). RNAseq was performed on a single experiment in triplicate. **(F-H)** DHT regulated genes (n = 471) were identified by quantifying mRNA expression in starved (vehicle; Ethanol 0.1 %) media and DHT induced media (P value ≤ 0.05, absolute log_2_ fold change > 1) (F, G). Out of 471 DHT regulated genes, 151 remain differentially expressed after Thio-2 treatment at 5 μM (F, H). Venn and volcano plots are shown. Horizontal dotted line indicates the significance threshold (P = 0.05). Vertical dotted line indicates the fold change threshold (absolute log_2_ fold change > 1).

Following the interrogation of our patient derived models, we explored the growth inhibitory effect of Thio-2 across multiple prostate cancer cell lines with varying levels of AR protein expression (Figure 6A, Supplementary Figure 6F). Although the AR positive cell line LNCaP was most sensitive to Thio-2 treatment, both the growth of AR positive (22Rv1) and AR negative (DU145 and PC3) cell lines was inhibited at higher Thio-2 concentrations, suggesting not all Thio-2 growth inhibitory effects are mediated through AR dependent mechanisms (Figure 6A). To further explore this, RNA-sequencing (RNA-seq) was performed in LNCaP to investigate the broader effects of Thio-2 (50 μM) on cellular pathways (Figure 6B). Six pathways were found to be significantly enriched after Thio-2 treatment; Thio-2 treatment suppressed important pathways implicated in prostate cancer biology including androgen response (NES -2.43, FDR < 0.01), E2F targets (NES -2.96, FDR < 0.01), G2M checkpoints (NES -2.59, FDR < 0.01), MYC targets V1 (NES -2.38, FDR < 0.01) and MYC targets V2 (NES -2.14, FDR < 0.01) (Figure 6C-D). Next, we investigated whether lower concentrations (5 μM) of Thio-2 are also sufficient to inhibit AR signaling and genome-wide AR binding in response to DHT (Figure 6E). The expression of 471 genes significantly (P ≤ 0.05, absolute log_2_ fold change > 1) changed in response to DHT (Figure 6F-G). Treatment with 5 μM Thio-2 led to a reduction in gene expression changes, with 151 (32%) of those 471 DHT-regulated genes remaining altered following DHT treatment (Figure 6F and H). Furthermore, AR chromatin immunoprecipitation demonstrated a reduction in genome wide AR binding (Supplementary Figure 7). Overall, these data suggest that Thio-2 impacts critical pathways, including AR signaling, involved in the development and progression of CRPC.

### The mechanism of action of Thio-2 is independent of BAG-1 isoform function

**Figure 7:**
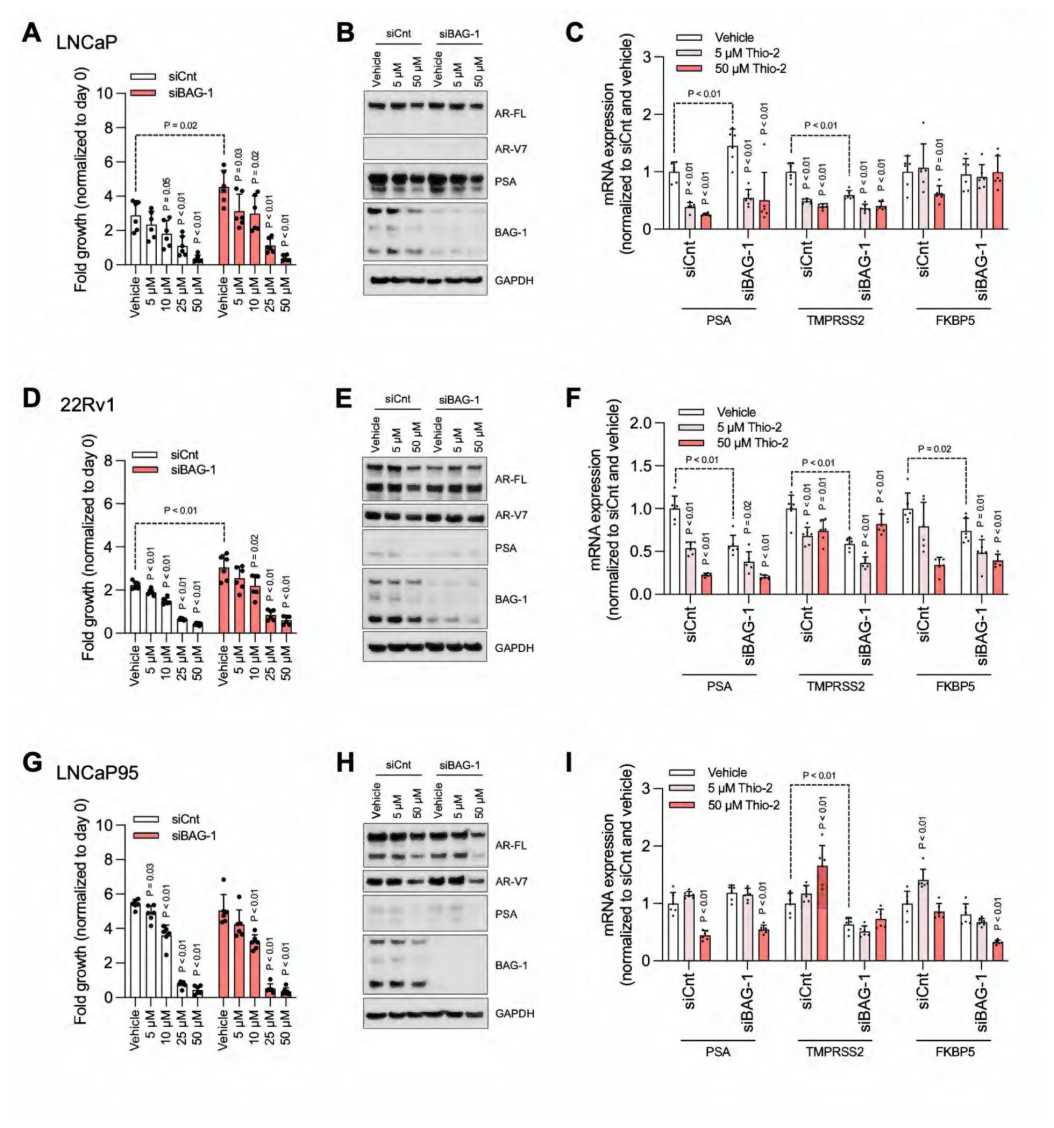
Thio-2 downregulates androgen receptor signaling and inhibits growth of prostate cancer cell lines through a BAG-1 independent mechanism. **(A, D and G)** LNCaP (A), 22Rv1 (D) and LNCaP95 (G) prostate cancer cells were transfected with 50nM of either control (siCnt; clear bars) or BAG-1 (siBAG-1; red bars) siRNA for 72 hours prior to treatment with vehicle (DMSO 0.1 %) or various concentrations (5, 10, 25 and 50 μM) of Thio-2 and growth was determined after 6 days by CellTiter-Glo® Luminescent Cell Viability Assay. Mean fold change in growth (compared to day 0) with standard deviation from a single experiment with six replicates is shown. P values were calculated for each condition compared to vehicle in siCnt and siBAG-1 cells, and between vehicle treated siCnt and siBAG-1 cells (dotted lines), using unpaired Student t-test. P values ≤ 0.05 are shown. **(B, E and H)** LNCaP (B) 22Rv1 (E) and LNCaP95 (H) prostate cancer cells were transfected with 50 nM of either siCnt or siBAG-1 siRNA for 55 hours prior to treatment with vehicle (DMSO 0.1 %) or various concentrations (5 and 50 μM) of Thio-2 for 17 hours (total 72 hours) and AR-FL, AR-V7, PSA, BAG-1 and GAPDH protein expression was determined. Single western blot is shown. **(C, F and I)** LNCaP (C), 22Rv1 (F) and LNCaP95 (I) prostate cancer cells were transfected with 50 nM of either siCnt or siBAG-1 siRNA for 55 hours prior to treatment with vehicle (DMSO 0.1 %) or various concentrations (5 and 50 μM) of Thio-2 for 17 hours (total 72 hours) and PSA, TMPRSS2 and FKBP5 mRNA expression was determined. Mean mRNA expression (normalized to average of GAPDH/B2M/HRPT1/RPLP0 and siCnt/vehicle; defined as 1) with standard deviation from a single experiment with six replicates is shown. P values were calculated for each condition compared to vehicle in siCnt and siBAG-1 cells, and between vehicle treated siCnt and siBAG-1 cells (dotted lines), using unpaired Student t-test. P values ≤ 0.05 are shown.

In light of Thio-2 being reported to inhibit BAG-1 isoform function, we next interrogated whether the observed mechanism of action of Thio-2 is dependent on the BAG-1 isoforms (15, 26, 53). BAG-1 isoform siRNA knockdown led to a small but significant increase in growth of LNCaP (P = 0.02, Student t-test) and 22Rv1 (P < 0.01, Student t-test), but not in LNCaP95 cells (Figure 7A, D and G). In addition, BAG-1 isoform siRNA knockdown had no effect on AR-FL, AR splice variant-7 (AR-V)7 or PSA protein expression in all three cell lines (Figure 7B, E and H). Furthermore, BAG-1 isoform siRNA knockdown did not consistently suppress downstream AR target genes (Figure 7C, F and I). However, as previously shown, Thio-2 significantly inhibited the growth of LNCaP, 22Rv1 and LNCaP95 (Figure 6A; Figure 7A, D and G). This was irrespective of BAG-1 isoform siRNA knockdown status, suggesting that its growth inhibitory effects are not BAG-1 isoform mediated (Figure 7A, D and G). In contrast to BAG-1 knockdown, Thio-2 suppressed AR target genes more consistently across all cell lines tested, independent of BAG-1 isoform expression (Figure 7C, F and I). To further validate these findings, we developed 22Rv1 and LNCaP95 BAG-1 isoform CRISPR knockout clones. BAG-1 isoform CRISPR knockout using three different guides in 22Rv1 led to a significant (all P < 0.01, Student t-test) increase in growth (Supplementary Figure 8A). In contrast, BAG-1 isoform CRISPR knockout using two different guides in LNCaP95 led to a significant (P = 0.04 and 0.03, Student t-test) decrease in growth (Supplementary Figure 8D). BAG-1 isoform CRISPR knockout did not consistently impact AR-FL or AR-V7 protein levels in these cell lines (Supplementary Figure 8B and E). In addition, BAG-1 isoform CRISPR knockout did not significantly suppress any AR target genes, with several of them significantly increasing in response to BAG-1 knockout (Supplementary Figure 8C and F). Consistent with our siRNA studies, in our CRISPR models, Thio-2 treatment significantly inhibited the growth, and suppressed AR target gene expression more consistently, with this appearing to be independent of BAG-1 isoform expression (Supplementary Figure 8A-F). Finally, as our RNA-seq data demonstrated that Thio-2 impacted other pathways including MYC targets V1 and V2, we explored the impact of Thio-2 treatment on C-MYC expression in our CP50 PDX-O and LNCaP95 BAG-1 isoform CRISPR knockout clones (Figure 6D, Supplementary Figure 9A-B). Consistent with our RNA-seq analyses, Thio-2 treatment decreased C-MYC protein expression in both models, and this was independent of BAG-1 isoform function (Supplementary Figure 9A-B). In addition, although Thio-2 treatment decreased C-MYC protein expression in LNCaP, LNCaP95, 22Rv1 and DU145 cell lines; C-MYC siRNA knockdown most consistently impacted the growth of DU145 cells, providing novel insights into the potential mechanism through which Thio-2 inhibits the growth of AR negative cell lines (Supplementary Figure 9C-F). Taken together, these studies confirm that Thio-2 inhibits AR signaling and decreases C-MYC expression to inhibit the growth of prostate cancer cell lines through a mechanism of action that, in these specific studies, appears to be independent of BAG-1 isoform function.

### The mechanism of action of Thio-2 in prostate cancer models may be mediated, in part, through a novel interaction with the androgen receptor N-terminus

Having demonstrated that Thio-2 inhibits AR signaling and the growth of prostate cancer models independent of BAG-1 isoform function, we investigated whether its mechanism of action may be mediated through the AR N-terminal domain (NTD) as it has previously been shown to inhibit AR NTD transactivation (15). EPI-001, which binds the AR NTD, leads to intensity changes in the NMR protein ^1^H-^15^N correlation spectra for the full-length AR NTD (residues 1-558) and chemical shift perturbations in the partially helical regions of a shorter transactivation unit 5 construct (residues 330-447) (Supplementary Figure 10A-B) (57, 58). Although less intense, Thio-2 demonstrated changes in the same regions of AR, suggesting that it may bind the AR through by a similar binding mechanism (Supplementary Figure 10C-D). Next, to further explore the impact on Thio-2 on AR transactivation, we interrogated the ability of Thio-2 to inhibit the transcriptional activity of both AR-FL and AR-V7. Thio-2 significantly inhibited the transactivation of the unstimulated and stimulated AR-FL, and the constitutively active AR-V7 (Supplementary Figure 11). In contrast, enzalutamide only inhibited the stimulated AR-FL, further supporting that Thio-2 may function through the AR NTD, independent of BAG-1, and distinct from current inhibitors of the Ligand Binding Domain (LBD), such as enzalutamide (Supplementary Figure 11). One important consideration is that Thio-2 does exhibit limited solubility, and although low micromolar concentrations at which growth inhibition and AR signaling suppression are observed can be achieved, those phenotypes seen at much higher concentrations should be interpreted with caution (Supplementary Figure 12). These studies suggest that the mechanism of action of Thio-2, and the associated phenotype observed, may be mediated, in part, through a novel interaction with the AR NTD, although other mechanisms of action are also likely and require further elucidation. These data support the interrogation of related compounds with improved drug-like properties, that elicit the same phenotype, as a novel therapeutic strategy for CRPC.

## Discussion

The clinical relevance of BAG-1 isoforms in prostate cancer has been studied extensively by immunohistochemistry. These studies have demonstrated increased BAG-1L protein expression as castration resistance develops (24, 25). In addition, multiple studies have demonstrated nuclear BAG-1 protein expression to associate with clinical benefit from AR-targeting therapies, and cytoplasmic BAG-1 protein expression to associate with benefit from radiotherapy in localized disease (15, 24, 25). To expand on these data and overcome the challenges associated with pre-analytical variables, we explored BAG-1 mRNA isoform expression and its associations with signaling pathways in two independent CRPC patient transcriptome cohorts (30–32, 59, 60). BAG-1 mRNA was highly expressed and associated with key pathways, such as MYC targets V1 and V2, E2F targets, IL6 JAK STAT signaling, DNA repair, PI3K AKT MTOR signaling, MTORC1 signaling and androgen response, that have been linked to the development and progression of CRPC (3, 61–64). Interestingly, BAG-1 mRNA isoform expression was only associated with androgen response in the SU2C/PCF cohort, which may, in part, be due to the fact that only around half of these patients had received an AR-targeting therapy compared to all of the patients in the ICR/RMH cohort (30–32). In addition, our mechanistic studies point to the fact that BAG-1 isoforms may not play a critical role in activation of the unstimulated AR, and this also needs to be considered. These findings are consistent with BAG-1 isoforms being multifunctional proteins that interact with a wide range of molecular targets to modulate multiple cellular processes supporting the development and progression of CRPC (8–10).

Critically, expansion of our previous druggability analyses demonstrate the BAG domain of BAG-1 isoforms to present a groove with geometric and physicochemical properties consistent with drug tractability that may be most suited to peptide or peptido-mimetic approaches as opposed to traditional small molecule inhibition. One concern with therapeutic inhibition of a multifunctional protein that regulates a plethora of physiological processes is ‘on-target’ toxicity (8–10). However, in contrast to previous studies that demonstrated BAG-1 homozygous knockout to be embryonically lethal, our alternative strategy identified BAG-1 knockout mice to be viable and fertile (15, 38). Interestingly, male mice had smaller prostates, and pregnant female mice had increased duration of pregnancy, decreased litter size, increased neonatal weight on day 1 and decreased neonatal survival on day 2, which may point to the known role of BAG-1 isoforms in hormone physiology (15, 17–19). However, it is important to note, that although this phenotype was observed, analyses of AR levels or localization, and signaling pathways, specifically in the mouse prostates identified no obvious underpinning mechanism. The difference in the phenotype observed when compared to previous studies may be a consequence of BAG-1 being located close to CHMP5 (opposite strand of chromosome 9), and the apparent down regulation of both genes, with CHMP5 being responsible for the phenotype observed (38, 52). A further consideration is that our strategy results in a small portion of the BAG-1 N-terminus being expressed that may confer functionality, as although C-terminal BAG domain mediated protein:protein interactions are reported to be critical for BAG-1 isoform function, the N-terminus of BAG-1L has been shown to play a role in regulating the AR (8–10, 15–23). Finally, our observations are consistent with the development of BAG-1 knockout embryonic stem cells that maintained pluripotency and the ability to differentiate, and studies of cancer cell lines where BAG-1 is rarely essential for cell survival (53, 65, 66).

A further consideration is that although BAG-1 mRNA associates with key signaling pathways implicated in the development and progression of prostate cancer in CRPC patient transcriptomes; this was not recapitulated in RNA-sequencing analyses of normal mouse prostates and LNCaP cells with BAG-1 knockout/knockdown, and no significant phenotype was observed with BAG-1 knockout/knockdown across multiple prostate cancer cell lines (3, 61–64). This is an important observation, as although one could postulate that BAG-1 function may be critical in the context of human disease complexity, which is very different to normal mouse prostate development and *in-vitro/vivo* prostate cancer cell line models, or BAG-1 knockout/knockdown may be incomplete, it also suggests that BAG-1 function may not be critical for prostate cancer cell survival and this requires careful consideration if targeting BAG-1 is to be pursued as a therapeutic target for this common disease.

Thio-2, a novel compound derived from Thioflavin S, has been predicted to bind the BAG domain of BAG-1 isoforms though *in-silico* docking experiments, and studies in melanoma, breast and prostate cancer cell lines, have suggested a reduction in binding of BAG-1 to its binding partners (such as HSC/P70, BRAF and AR), to inhibit AR, MEK and AKT signaling (15, 26, 53). Intriguingly, Thio-2 inhibits AR signaling, and other important pathways, such as E2F targets, G2M checkpoints, MYC targets V1 and MYC targets V2, implicated in the development and progression of CRPC, to inhibit the growth of treatment resistant prostate cancer cell lines and patient derived prostate cancer models. It is therefore unsurprising that, although LNCaP cells which are AR positive were the most sensitive to Thio-2 treatment, higher concentrations of Thio-2 inhibited the growth of AR negative cell lines, as this is likely mediated through AR independent molecular mechanisms. This was further supported by the demonstration that, although Thio-2 decreased C-MYC protein expression in multiple prostate cancer models, AR negative cell lines were more sensitive to C-MYC protein knockdown, suggesting Thio-2 may inhibit the growth of AR negative cell lines through a C-MYC mediated mechanism.

Interestingly, enzalutamide maintained some growth inhibitory effects across two PDX organoid (PDX-O) models (CP50 and CP89) tested whilst Thio-2 demonstrated activity across all three PDX-O tested. Although these PDX models were developed from metastatic lymph node biopsies of patients with CRPC, we have previously demonstrated that AR-V7 protein expression, a common mechanism of resistance to AR directed therapies, increases in response to castration and is suppressed by testosterone (55, 56). Consistent with this, our PDX models (CP50, CP89 and CP142; maintained in intact mice) have no/low levels of AR-V7 protein expression, and when grown as PDX-Os *in-vitro* demonstrate no measurable AR-V7 protein expression. This raises the possibility of re-sensitization to therapies targeting the AR which will be important to further understand mechanistically, especially in the context of ongoing studies exploring bipolar androgen therapy as a therapeutic approach in CRPC (67).

Importantly, neither our BAG-1 isoform knockdown or knockout studies recapitulated or rescued the Thio-2 phenotype observed, suggesting that the mechanism of action of Thio-2 is independent of BAG-1 function, in those specific molecular backgrounds studied. Interestingly, siRNA mediated BAG-1 isoform knockdown increased growth in LNCaP and 22Rv1 cells, but not LNCaP95 cells. Furthermore, CRISPR mediated BAG-1 isoform knockout increased growth in 22Rv1 cells but decreased growth in LNCaP95 cells. Although BAG-1 isoform knockdown or knockout had limited consistent anti-tumor efficacy in the CRPC models studied, it is important to note that this may be context dependent. In addition, the generation of CRISPR mediated BAG-1 isoform knockout clones from heterogenous cell populations, such as LNCaP95, may explain some of the differential effects seen between knockdown and knockout approaches (68). It will be important to consider these results and the implications for targeting BAG-1 isoforms in prostate and other cancers. It will be critical to further understand the role of BAG-1 isoforms in the context of the activated AR in CSPC, different molecular backgrounds, and different diseases, to fully determine whether BAG-1 isoform targeting should be pursued for anti-cancer drug discovery efforts (15, 17, 18). This is further emphasized by studies in multiple breast cancer cell lines that have demonstrated differential response in growth to BAG-1 knockdown or knockout (53, 69).

Consistent with these findings, we identified that Thio-2 may bind directly to the AR NTD through a similar mechanism to that of EPI-001, blocking both AR-FL and AR-V7 transactivation in reporter based assays, suggesting, in part, a novel mechanism of Thio-2 action in CRPC (58). This may not be unexpected as cell-based assays investigating the impact of BAG-1L on AR transactivation have commonly utilized the AR NTD (15, 18). However, one consideration is that Thio-2 does exhibit limited solubility, and although low micromolar concentrations at which growth inhibition and AR signaling suppression can be achieved, one should exercise caution when interpreting those phenotypes observed at much higher concentrations, and when considering the *in-vivo* analyses as both systemic and tumor Thio-2 concentrations were not determined. Furthermore, treatment with Thio-2 led to a reduction in heart weight *in-vivo*, and although no other significant sequelae were identified, treatment was relatively short-term, and this would need to be considered if more drug-like compounds were to be pursued. Importantly, the attractive phenotype exhibited supports the development of similar, more drug-like compounds, such as A4B17 and X15695, as a novel therapeutic approach for CRPC (27–29). However, it will be important to understand whether these compounds share a similar mechanism of action and elicit the same phenotype in CRPC models (27, 28). Importantly, although the AR NTD remains a challenging drug target, these data highlight the clinical potential of therapies targeting the AR NTD which are currently undergoing clinical evaluation in CRPC (70, 71).

In summary, we demonstrate the clinical relevance of BAG-1 isoform expression in CRPC and identify BAG-1 isoforms to be tractable but challenging drug target with knockout mouse models supporting the tolerability of blocking BAG-1 function therapeutically. Despite these promising data, genomic abrogation of BAG-1 isoforms appeared to have limited anti-tumor efficacy in the CRPC models studied suggesting protein redundancy. Importantly, although probably independent of BAG-1 isoform function, Thio-2 suppressed AR signaling and other key prostate cancer signaling pathways to inhibit the growth of CRPC models. This observed phenotype appeared to be, at least in part, mediated through direct binding to the AR NTD. These data support further development of similar, more drug-like compounds, such as A4B17 and X15695, as novel therapeutics for CRPC, and highlight the clinical potential of treatments that block persistent AR signaling which are currently undergoing clinical evaluation in CRPC (27–29, 70, 71).

## Supplementary Material

Figure S1

Figure S2

Figure S3

Figure S4

Figure S5

Figure S6

Figure S7

Figure S8

Figure S9

Figure S10

Figure S11

Figure S12

Supplementary methods

Table S1

Table S2

Table S3

Table S4

Table S5

Table S6

Table S7

## Data Availability

The cell line and mouse prostate RNA-sequencing data will be available under accession number PRJEB66442 from European Nucleotide Archive at time of publication.
