# Supplementary material for: Thio-2 inhibits key signaling pathways required for the development and progression of castration resistant prostate cancer
Source: Mol Cancer Ther. Author manuscript; Available in PMC 2024 Jun 5. (PMC11148553; doi:10.1158/1535-7163.MCT-23-0354)
Supplement: Figure S1 [file EMS194541-supplement-Figure_S1.pdf]

Supplementary Figure 1

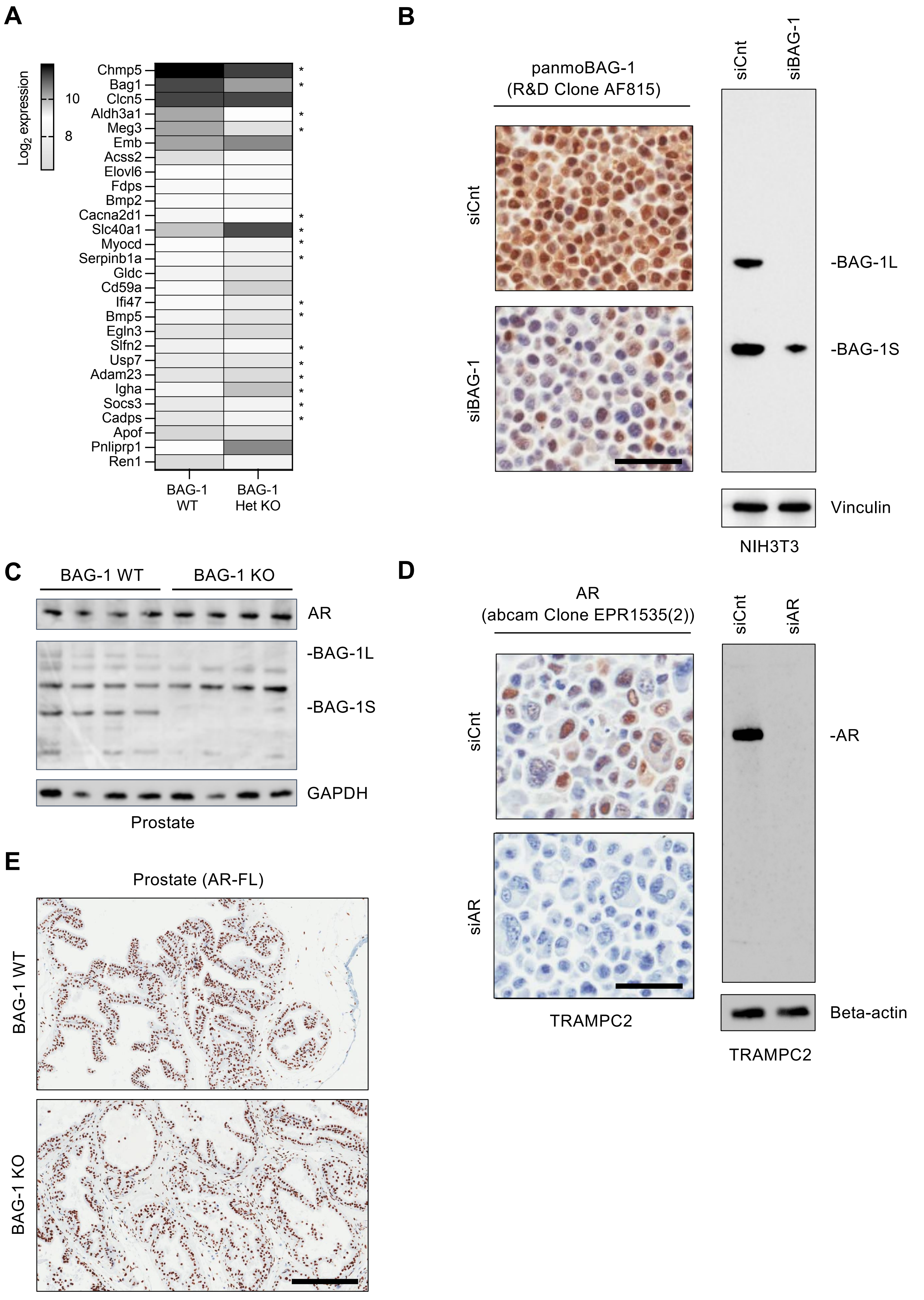

Supplementary Figure 1: Development and characterization of BAG-1 knockout mice.

(A) Microarray data from BAG-1 wildtype (WT) and exon 1 BAG-1 heterozygous knockout (Het KO) mouse prostates following up to 12-weeks castration demonstrating down-regulation of BAG-1 and CHMP5. Log_2 expression of those genes most altered is shown. P values were calculated for differences in gene expression between BAG-1 WT and BAG-1 Het KO using unpaired Student t-test. P values ≤ 0.05 are shown (*). **(B)** Representative western blot and immunohistochemistry of BAG-1 detection using a pan-mouse-BAG-1 (panmoBAG-1) antibody in NIH3T3 cells treated with control (siCnt) or BAG-1 (siBAG-1) siRNA. Scale bar, 50 µm. **(C)** Western blots on lysates of prostates from BAG-1 KO (n = 4) and BAG-1 wildtype (WT; n = 4) male mice were analyzed for BAG-1, AR and GAPDH protein levels. Single western blot is shown. **(D)** Representative western blot and immunohistochemistry of AR detection using a mouse/human AR-FL antibody in TRAMPC2 cells treated with control (siCnt) or AR (siAR) siRNA. Scale bar, 50 µm. **(E)** Prostates from BAG-1 knockout (BAG-1 KO) mouse strain Bag1tm1a(EUCOMM)Hmgu and BAG-1 WT male mice were analyzed for mouse AR protein (immunohistochemistry) levels. Representative micrographs of AR detection in mouse prostates by mouse/human AR antibody IHC are shown. Scale bar, 200 µm.
