# Supplementary material for: Thio-2 inhibits key signaling pathways required for the development and progression of castration resistant prostate cancer
Source: Mol Cancer Ther. Author manuscript; Available in PMC 2024 Jun 5. (PMC11148553; doi:10.1158/1535-7163.MCT-23-0354)
Supplement: Figure S2 [file EMS194541-supplement-Figure_S2.pdf]

Supplementary Figure 2

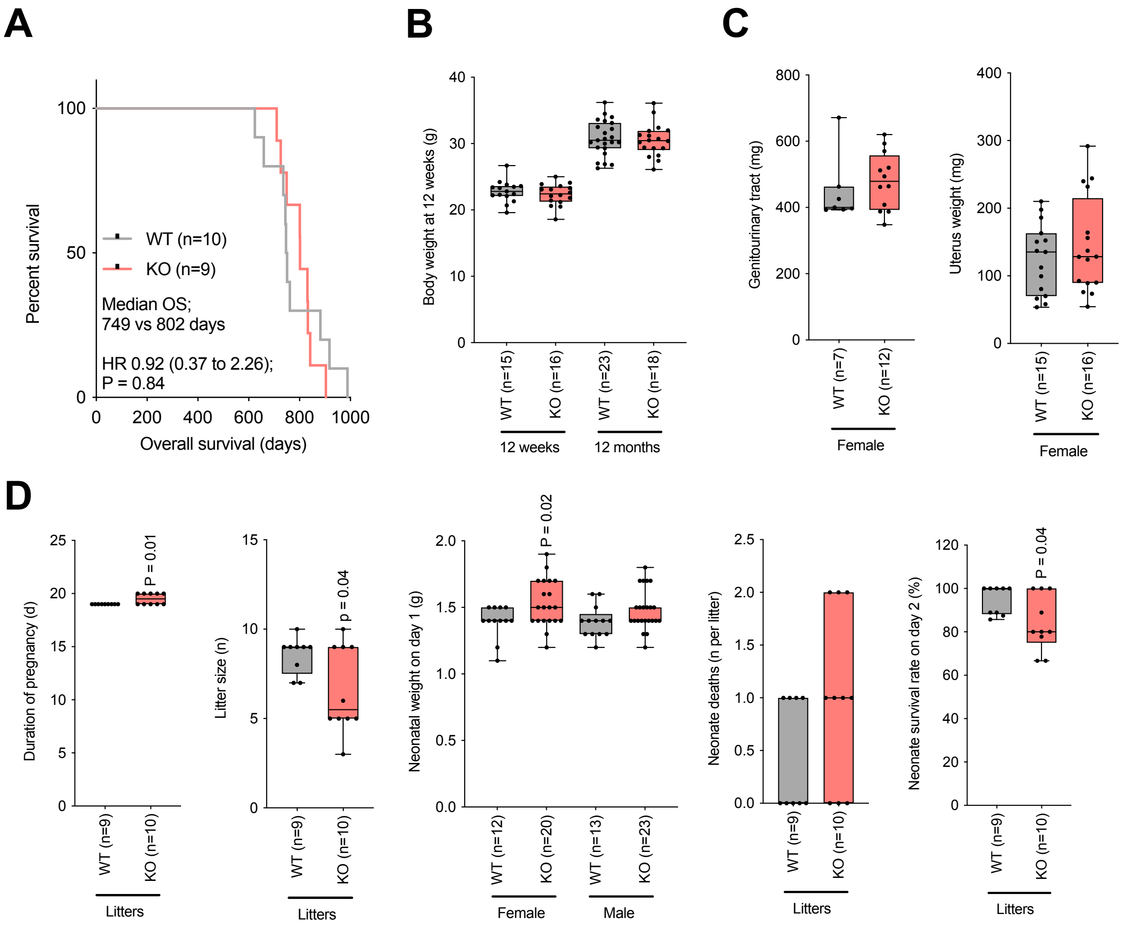

Supplementary Figure 2: BAG-1 knockout female mice are fertile and viable with reduced gestation.

(A) Kaplan-Meier curves of overall survival (OS) of BAG-1 KO (red line; n=9) and BAG-1 WT (gray line; n=10) female mice from birth. Median OS, hazard ratio (HR) with 95% confidence intervals and P values for univariate Cox survival model are shown. **(B)** The body weight of BAG-1 KO (red bars) and BAG-1 WT (gray bars) female mice at 12 weeks and 12 months was determined. Median body weight with interquartile range, and smallest and largest value, is shown. P values were calculated for BAG-1 KO compared with BAG-1 WT mice using unpaired Student t-test. P values $\leq$ 0.05 are shown. **(C)** The weight of the genitourinary tract and uterus from BAG-1 KO (red bars) and BAG-1 WT (gray bars) female mice at 3 months and older was determined. Median weight with interquartile range, and smallest and largest value, is shown. P values were calculated for BAG-1 KO compared with BAG-1 WT mice using unpaired Student t-test. P values $\leq$ 0.05 are shown. **(D)** The duration of pregnancy, litter size, neonatal weight (on day 1), neonatal deaths (per litter) and neonatal survival rate (on day 2) for female BAG-1 KO (red bars) and BAG-1 WT (gray bars) mice was determined. Median values with interquartile range, and smallest and largest value, is shown. P values were calculated for BAG-1 KO compared with BAG-1 WT mice using unpaired Student t-test. P values $\leq$ 0.05 are shown.
