# Supplementary material for: Thio-2 inhibits key signaling pathways required for the development and progression of castration resistant prostate cancer
Source: Mol Cancer Ther. Author manuscript; Available in PMC 2024 Jun 5. (PMC11148553; doi:10.1158/1535-7163.MCT-23-0354)
Supplement: Figure S3 [file EMS194541-supplement-Figure_S3.pdf]

Supplementary Figure 3

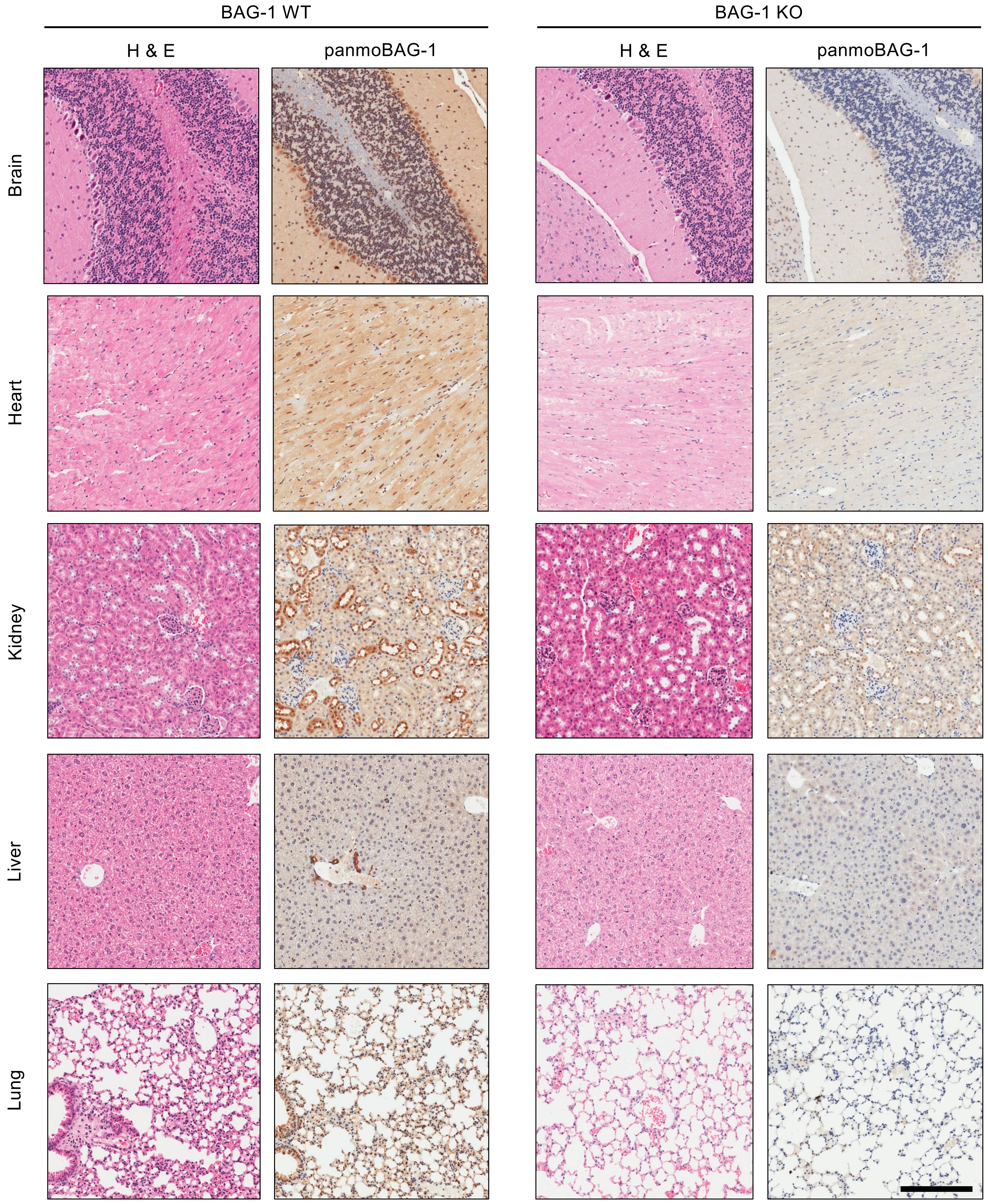

Supplementary Figure 3: Mouse organ hematoxylin and eosin, and BAG-1 immunohistochemistry, in BAG-1 knockout mice.

Organs (brain, heart, kidney, liver, and lung) from BAG-1 KO and BAG-1 WT mice at 4 months and older were analyzed by hematoxylin and eosin (H & E) and for pan-mouse-BAG-1 (panmoBAG-1) protein (IHC) levels. Representative micrographs of H & E, and BAG-1 detection, in mouse organs by panmoBAG-1 antibody IHC are shown. Scale bar, 200 μm.
