# Supplementary material for: Thio-2 inhibits key signaling pathways required for the development and progression of castration resistant prostate cancer
Source: Mol Cancer Ther. Author manuscript; Available in PMC 2024 Jun 5. (PMC11148553; doi:10.1158/1535-7163.MCT-23-0354)
Supplement: Figure S4 [file EMS194541-supplement-Figure_S4.pdf]

Supplementary Figure 4

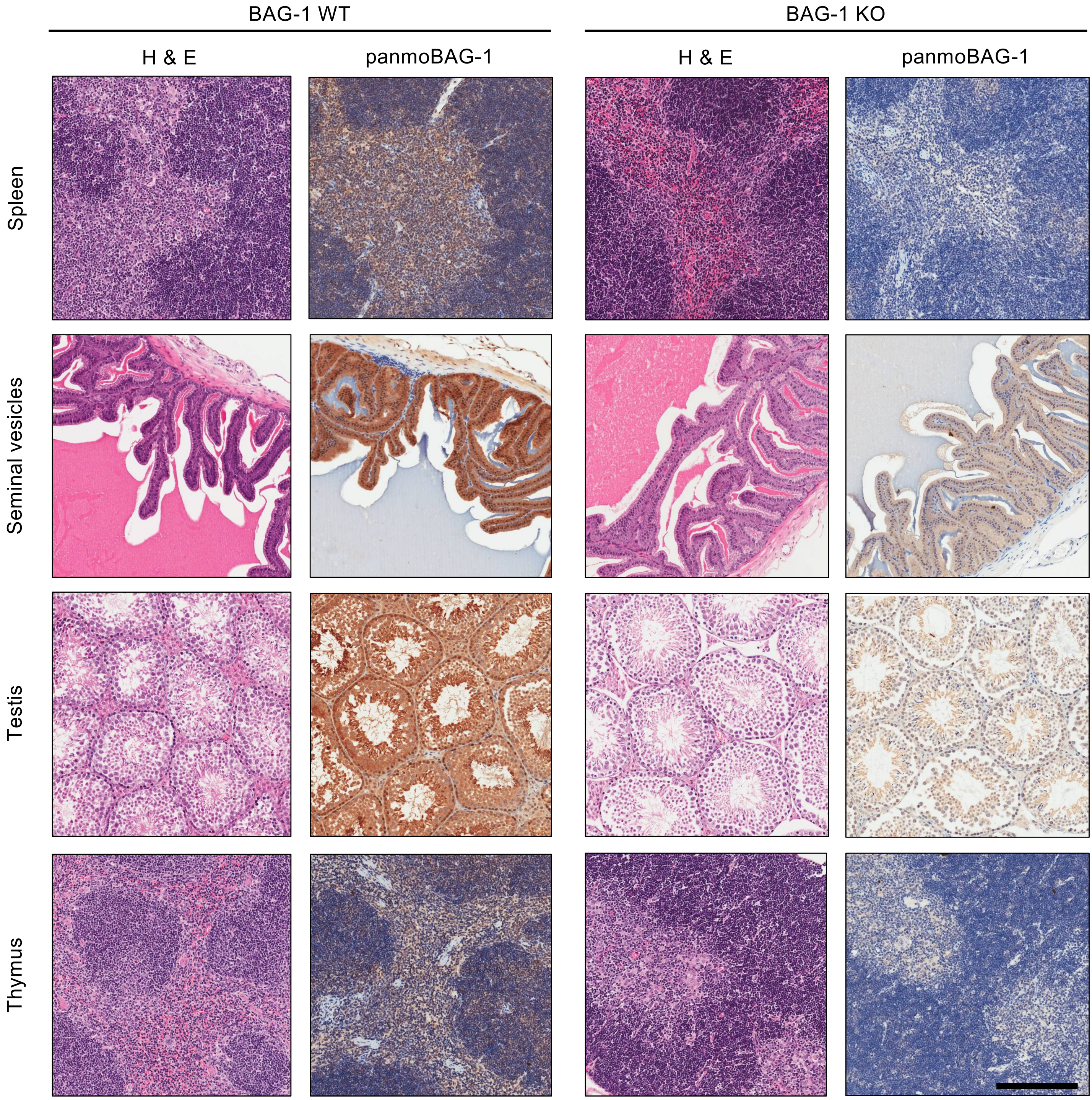

Supplementary Figure 4: Mouse organ hematoxylin and eosin, and BAG-1 immunohistochemistry, in BAG-1 knockout mice.

Organs (spleen, seminal vesicles, testis, and thymus) from BAG-1 KO and BAG-1 WT mice at 3 months and older were analyzed by H & E and for pan-mouse-BAG-1 (panmoBAG-1) protein (IHC) levels. Representative micrographs of H & E, and BAG-1 detection, in mouse organs by panmoBAG-1 antibody IHC are shown. Scale bar: 200 µm.
