# Supplementary material for: Thio-2 inhibits key signaling pathways required for the development and progression of castration resistant prostate cancer
Source: Mol Cancer Ther. Author manuscript; Available in PMC 2024 Jun 5. (PMC11148553; doi:10.1158/1535-7163.MCT-23-0354)
Supplement: Figure S5 [file EMS194541-supplement-Figure_S5.pdf]

Supplementary Figure 5

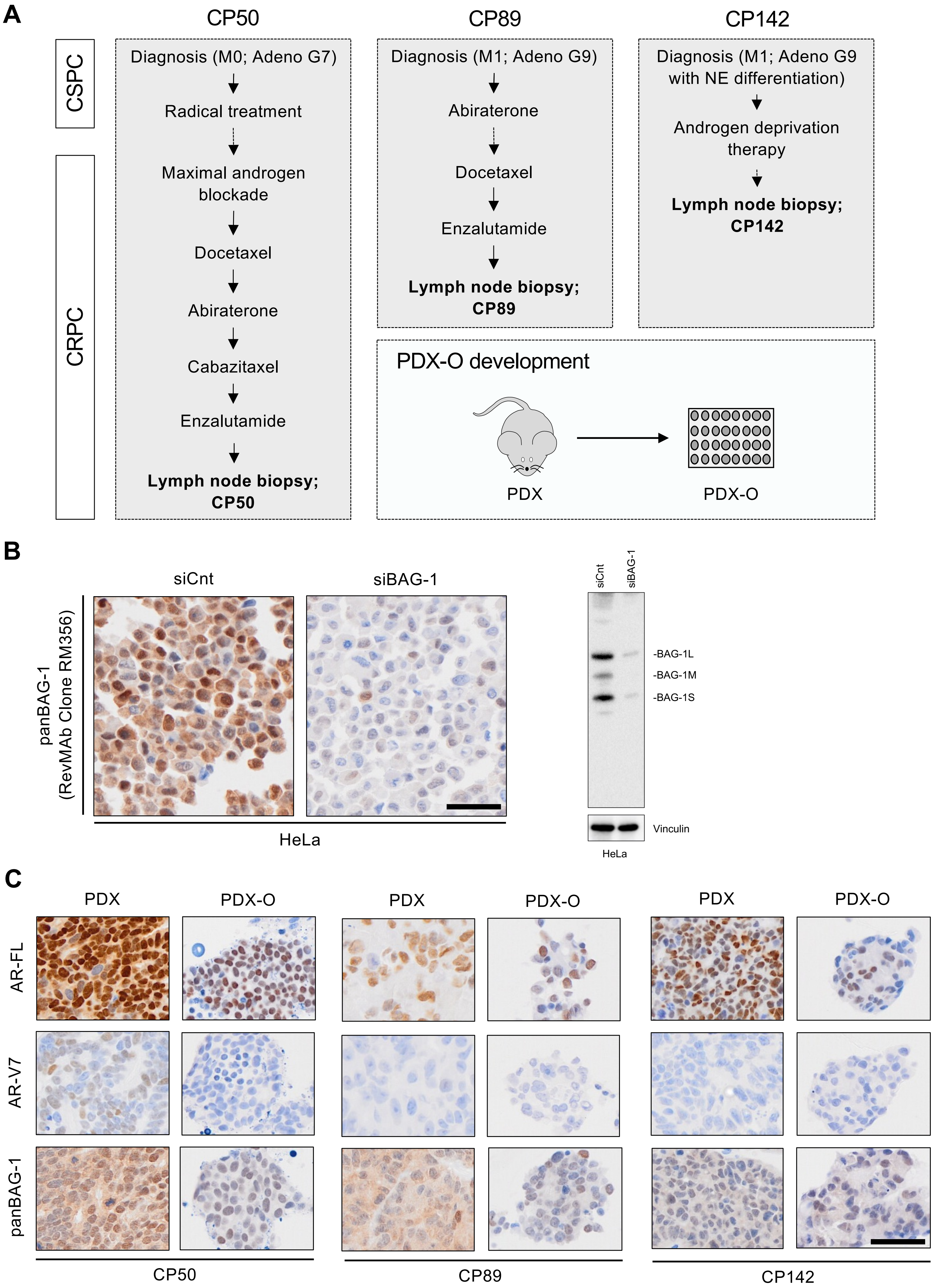

Supplementary Figure 5: Development of castration resistant prostate cancer patient derived xenograft organoids

(A) Patient derived xenografts (PDX) CP50, CP89 and CP142 were derived from metastatic lymph node biopsies of patients with castration resistant prostate cancer (CRPC) treated with multiple standard treatments for castration sensitive prostate cancer (CSPC) and CRPC. Staging (metastasis (M) yes (1) or no (0)) and histology (Gleason score (G) with or without neuroendocrine differentiation (NE) at diagnosis, treatments received for CSPC and CRPC, and timing of metastatic biopsy are shown. Once PDX were established (> 3 passages), PDX-organoids (PDX-O) were derived from fresh PDX tumors and plated in 96 well plates for drug treatment experiments presented. **(B)** Representative western blot and immunohistochemistry (IHC) of pan-BAG-1 (panBAG-1) detection using a pan-BAG-1 antibody in HeLa cells treated with control (siCnt) or BAG-1 (siBAG-1) siRNA. Scale bar, 50 µm. **(C)** CP50, CP89 and CP142 PDX tumors and derived PDX-Os were formalin-fixed paraffin-embedded, and androgen receptor (AR-FL), androgen receptor splice variant-7 (AR-V7) and panBAG-1 immunohistochemistry (IHC) performed. Representative micrographs of AR-FL, AR-V7 and pan BAG-1 (panBAG-1) detection by IHC are shown. Scale bar, 50 µm.
