# Supplementary material for: Thio-2 inhibits key signaling pathways required for the development and progression of castration resistant prostate cancer
Source: Mol Cancer Ther. Author manuscript; Available in PMC 2024 Jun 5. (PMC11148553; doi:10.1158/1535-7163.MCT-23-0354)
Supplement: Figure S6 [file EMS194541-supplement-Figure_S6.pdf]

Supplementary Figure 6

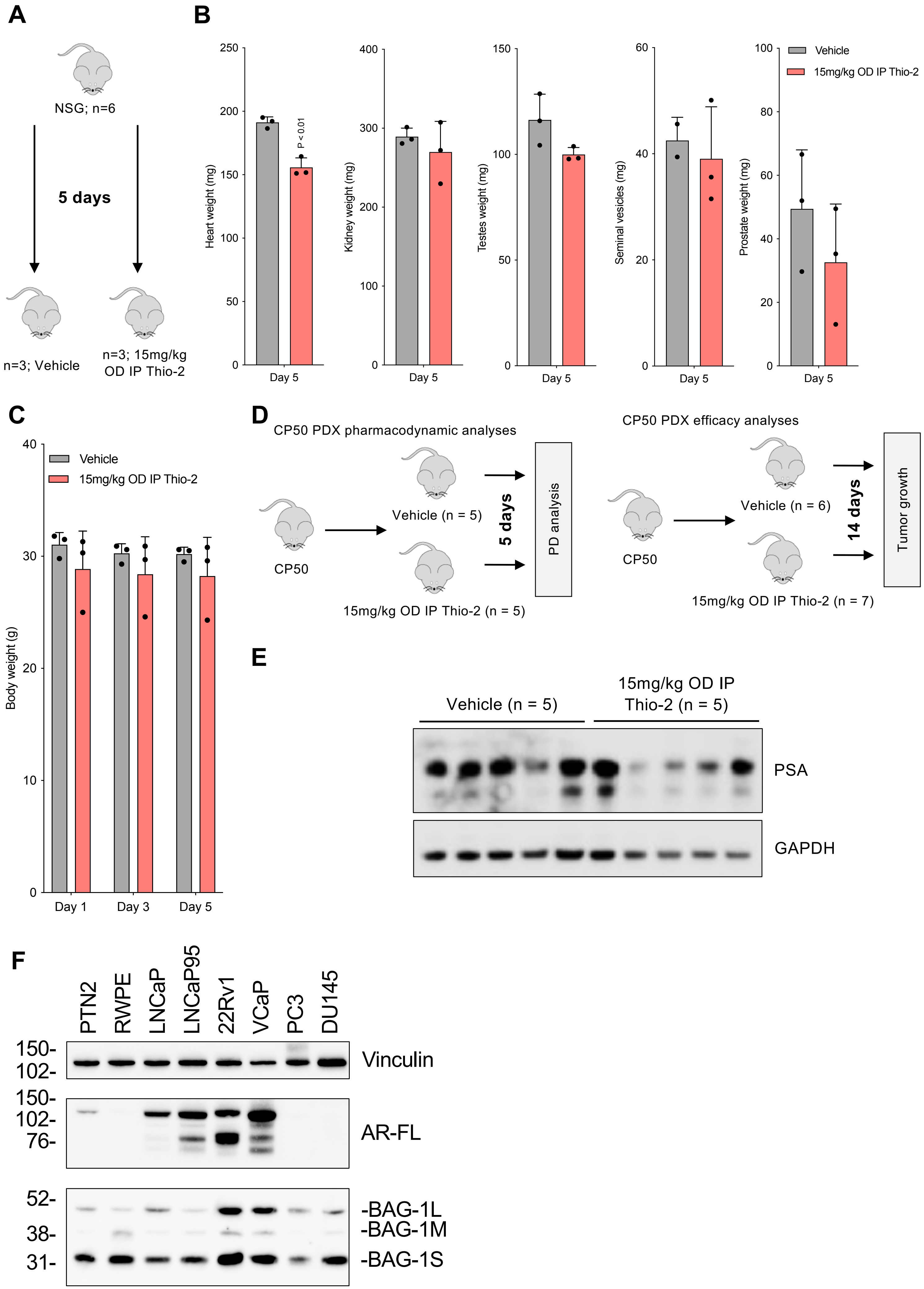

Supplementary Figure 6: *In-vivo* treatment of patient derived model of lethal prostate cancer demonstrates Thio-2 to be well tolerated with associated impact on AR signaling and growth.

(A-C) The impact of vehicle (5 % DMSO in 10 % (w/v) HBC in 0.9 % saline) and 15 mg/kg Thio-2 once daily (OD) intraperitoneal (IP) on NSG mice (n=3 per arm; A) on heart, kidney, testes, seminal vesicles and prostate weights (B), and total body weight (C) was determined. P values were calculated for vehicle compared to 15 mg/kg Thio-2 OD IP using unpaired Student t-test. P values ≤ 0.05 are shown. **(D)** Schematic overview of experimental design using patient derived xenograft (PDX) CP50 that was developed from a lymph node biopsy from a patient who had progressed through all standard of care treatments for castration resistant prostate cancer. Once CP50 PDX tumor volume reached 300 mm^3 treatment, 15 mg/kg Thio-2 or vehicle OD IP was commenced for 5 (pharmacodynamic analyses) or 14 days (efficacy analyses). **(E)** The effect of 15 mg/kg Thio-2 OD IP compared to vehicle on PSA and GAPDH protein expression in CP50 PDX tumors was determined at 5 days. **(F)** AR-FL, BAG-1 and Vinculin protein expression was determined across multiple prostate cancer cell lines. Single western blot is shown.
