# Supplementary material for: Thio-2 inhibits key signaling pathways required for the development and progression of castration resistant prostate cancer
Source: Mol Cancer Ther. Author manuscript; Available in PMC 2024 Jun 5. (PMC11148553; doi:10.1158/1535-7163.MCT-23-0354)
Supplement: Figure S7 [file EMS194541-supplement-Figure_S7.pdf]

Supplementary Figure 7

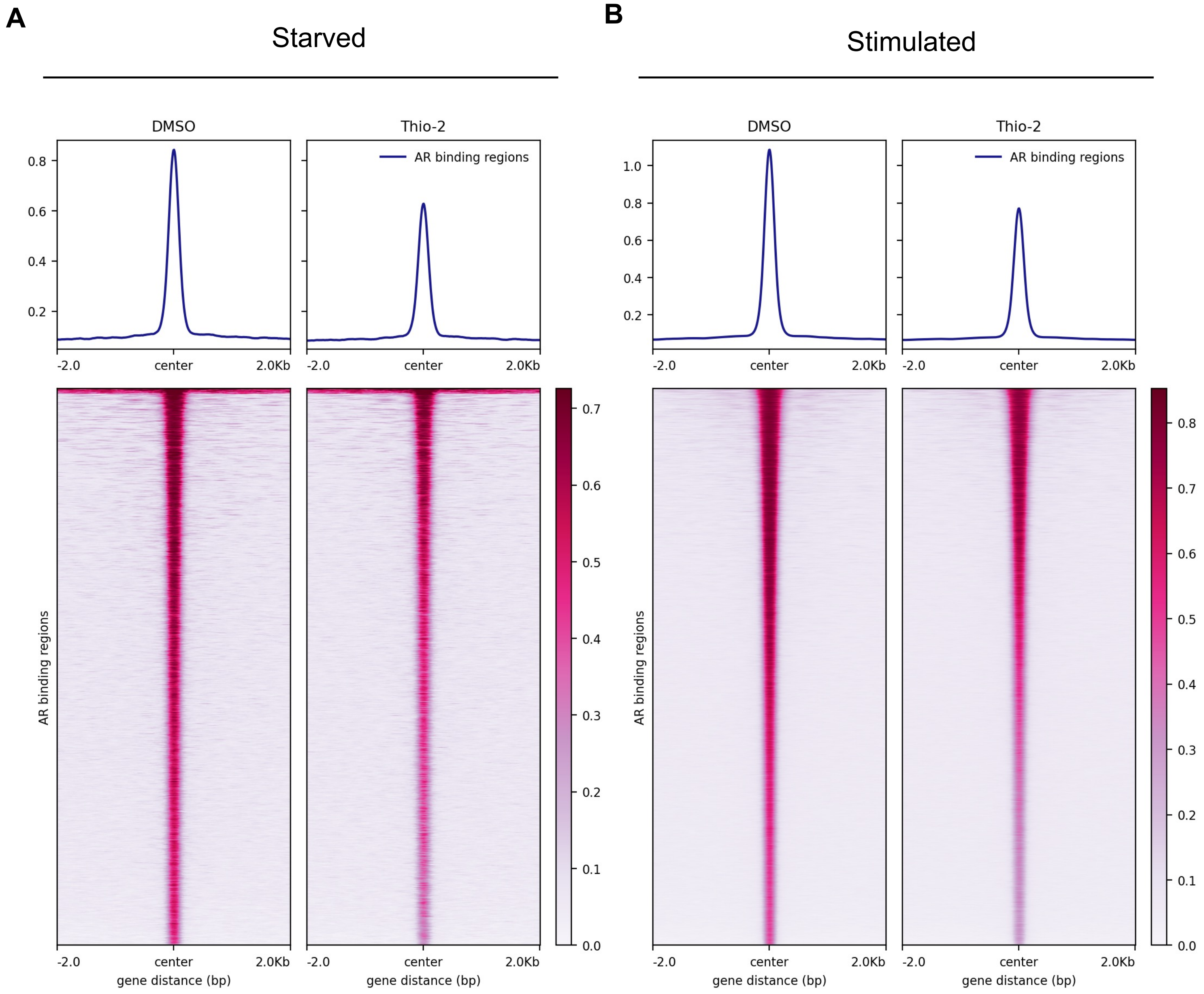

Supplementary Figure 7: Thio-2 reduces genome-wide androgen receptor binding in LNCaP prostate cancer cells.

LNCaP cells were grown in starved media (10 % charcoal stripped serum) for 72 hours, following 1 hour pre-treatment with vehicle or 5 µM Thio-2. Cells were subsequently treated with vehicle (Ethanol 0.1%) or 10 nM dihydrotestosterone (DHT) for 16 hours (17 hours total treatment). Chromatin immunoprecipitation sequencing (ChIP-seq) was performed on a single experiment in triplicate. Peak density histograms and heatmaps showing AR binding regions from ChIP-seq analysis for vehicle (DMSO 0.1 %) and 5 µM Thio-2 treated samples are shown. For starved (Ethanol 0.1 %) and stimulated (10 nM DHT) samples, AR binding within a 4 kilobases window is shown for pooled peak summits from both vehicle (DMSO 0.1 %) and 5 µM Thio-2 treated samples. Color bars show relative signal intensity.
