# Supplementary material for: Thio-2 inhibits key signaling pathways required for the development and progression of castration resistant prostate cancer
Source: Mol Cancer Ther. Author manuscript; Available in PMC 2024 Jun 5. (PMC11148553; doi:10.1158/1535-7163.MCT-23-0354)
Supplement: Figure S8 [file EMS194541-supplement-Figure_S8.pdf]

Supplementary Figure 8

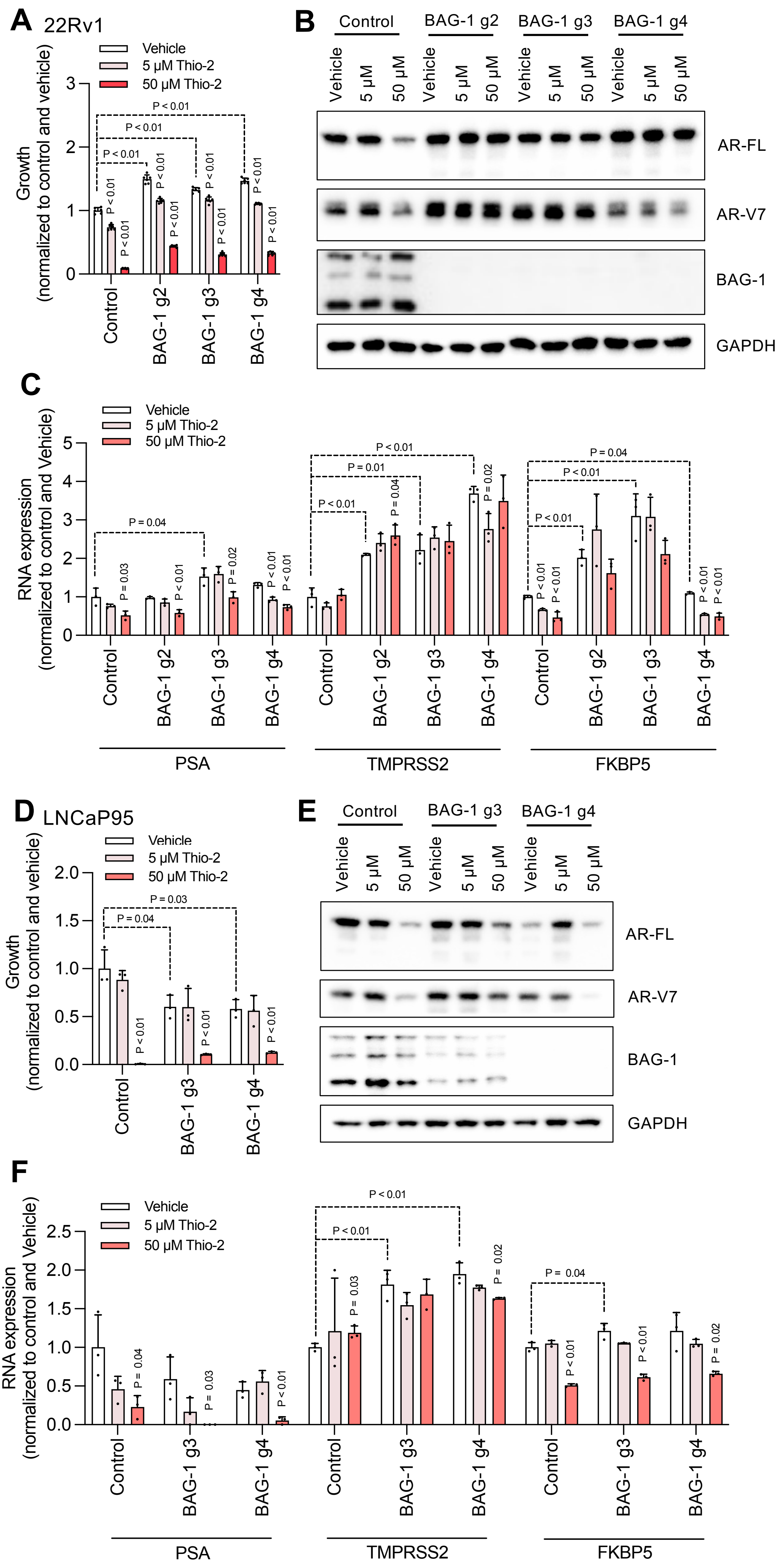

Supplementary Figure 8: Thio-2 downregulates androgen receptor signaling and inhibits growth of prostate cancer cell lines through a BAG-1 independent mechanism.

(A, D) BAG-1 knockout CRISPR/Cas9 clones were developed in 22Rv1 (A) and LNCaP95 (D) prostate cancer cells. Control (Cas9) and three BAG-1 knockout (guide 2, g2; guide 3, g3; guide 4, g4) were used for transfection and single cell derived 22Rv1 (control, g2, g3, g4) and LNCaP95 (control, g3, g4) clones were selected. Clones were treated with vehicle (DMSO 0.1 %) or various concentrations (5 and 50 µM) of Thio-2 and growth was determined after 6 days by CellTiter-Glo® Luminescent Cell Viability Assay. Mean growth (normalized to vehicle treated control clone; defined as 1) with standard deviation from a single experiment with six replicates is shown. P values were calculated for each condition compared to vehicle for each individual guide, and between vehicle treated control and BAG-1 guides (dotted lines), using unpaired Student t-test. P values ≤ 0.05 are shown. **(B, E)** 22Rv1 (B) and LNCaP95 (E) clones were treated with vehicle (DMSO 0.1 %) or various concentrations (5 and 50 µM) of Thio-2 for 17 hours and AR-FL, AR-V7, BAG-1 and GAPDH protein expression was determined. Single western blot representative of three is shown. **(C, F)** 22Rv1 (C) and LNCaP95 (F) clones were treated with vehicle (DMSO 0.1 %) or various concentrations (5 and 50 µM) of Thio-2 for 17 hours and PSA, TMPRSS2 and FKBP5 mRNA expression was determined. Mean mRNA expression (normalized to average of GAPDH/B2M/HRPT1/RPLP0 and vehicle treated control clone; defined as 1) with standard deviation from a single experiment with three replicates is shown. P values were calculated for each condition compared to vehicle for each individual guide, and between vehicle treated control and BAG-1 guides (dotted line), using unpaired Student t-test. P values ≤ 0.05 are shown.
