# Supplementary material for: Thio-2 inhibits key signaling pathways required for the development and progression of castration resistant prostate cancer
Source: Mol Cancer Ther. Author manuscript; Available in PMC 2024 Jun 5. (PMC11148553; doi:10.1158/1535-7163.MCT-23-0354)
Supplement: Figure S9 [file EMS194541-supplement-Figure_S9.pdf]

Supplementary Figure 9

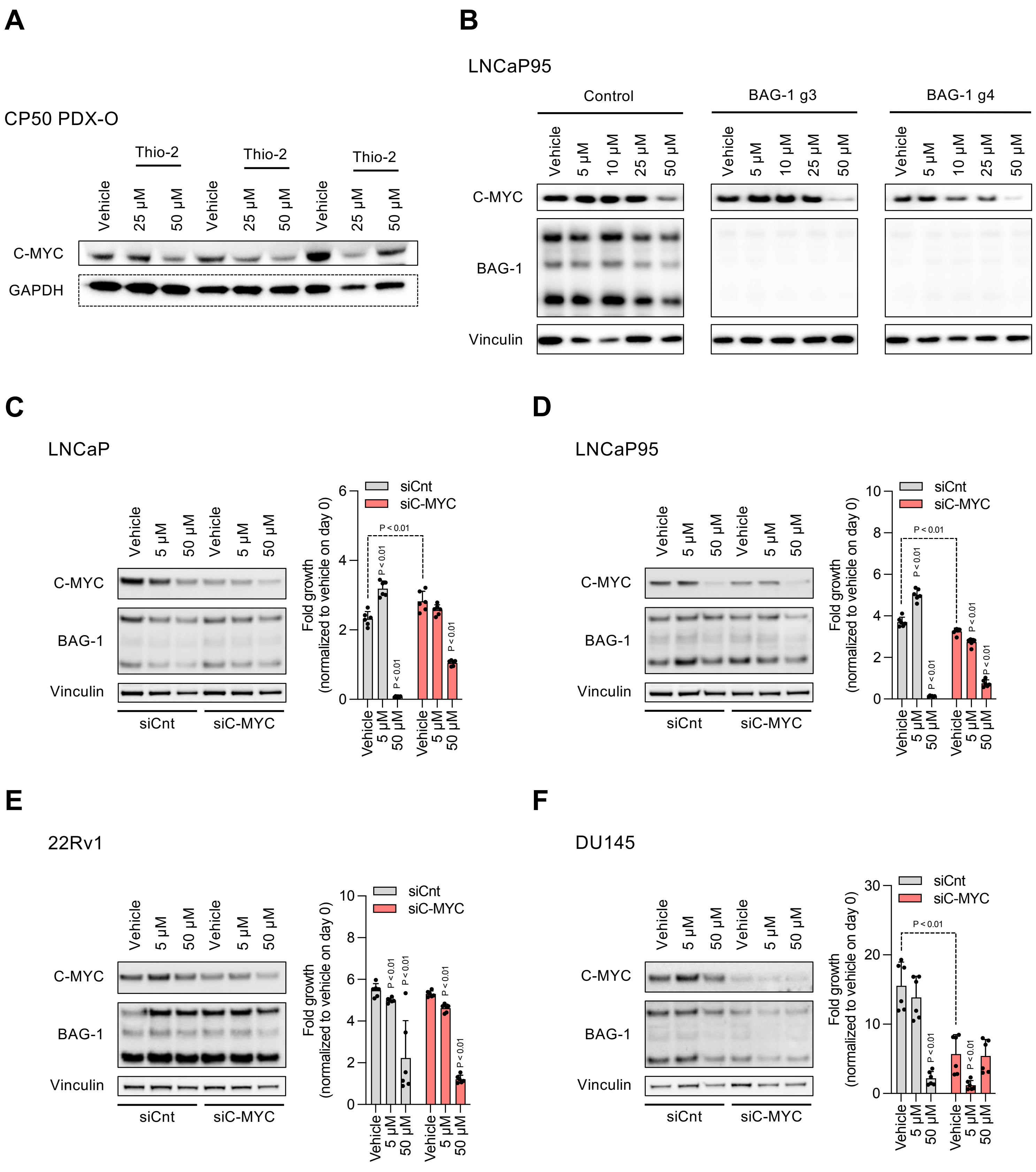

Supplementary Figure 9: Thio-2 downregulates C-MYC expression through a BAG-1 independent mechanism.

(A) CP50 PDX-O were treated with vehicle (DMSO 0.1 %) or various concentrations (25 and 50 μM) of Thio-2 for 17 hours. The effect of each condition on C-MYC and GAPDH (shown in figure 5C) protein expression was determined. Single western blot with triplicates is shown. **(B)** LNCaP95 clones were treated with vehicle (DMSO 0.1 %) or various concentrations (5, 10, 25 and 50 μM) of Thio-2 for 17 hours and C-MYC, BAG-1 and Vinculin protein expression was determined. Single western blot representative of three is shown. **(C-F)** LNCaP (C), LNCaP95 (D), 22Rv1 (E) and DU145 (F) prostate cancer cells were transfected with 50 nM of either control (siCnt) or C-MYC (siC-MYC) siRNA for 55 hours prior to treatment with vehicle (DMSO 0.1 %) or various concentrations (5 and 50 μM) of Thio-2 for 17 hours (total 72 hours) and C-MYC, BAG-1 and Vinculin protein expression was determined. Single western blot is shown. In addition, the impact of 50 nM siCnt (gray bars) and siC-MYC (red bars) for 72 hours prior to treatment with vehicle (DMSO 0.1 %) or various concentrations (5 and 50 μM) of Thio-2 on growth was determined after 6 days by CellTiter-Glo® Luminescent Cell Viability Assay. Mean fold change in growth (compared to day 0) with standard deviation from a single experiment with six replicates is shown. P values were calculated for each condition compared to vehicle in siCnt and siC-MYC cells, and between vehicle treated siCnt and siC-MYC cells (dotted lines), using unpaired Student t-test. P values ≤ 0.05 are shown.
