# Supplementary material for: Thio-2 inhibits key signaling pathways required for the development and progression of castration resistant prostate cancer
Source: Mol Cancer Ther. Author manuscript; Available in PMC 2024 Jun 5. (PMC11148553; doi:10.1158/1535-7163.MCT-23-0354)
Supplement: Figure S10 [file EMS194541-supplement-Figure_S10.pdf]

Supplementary Figure 10

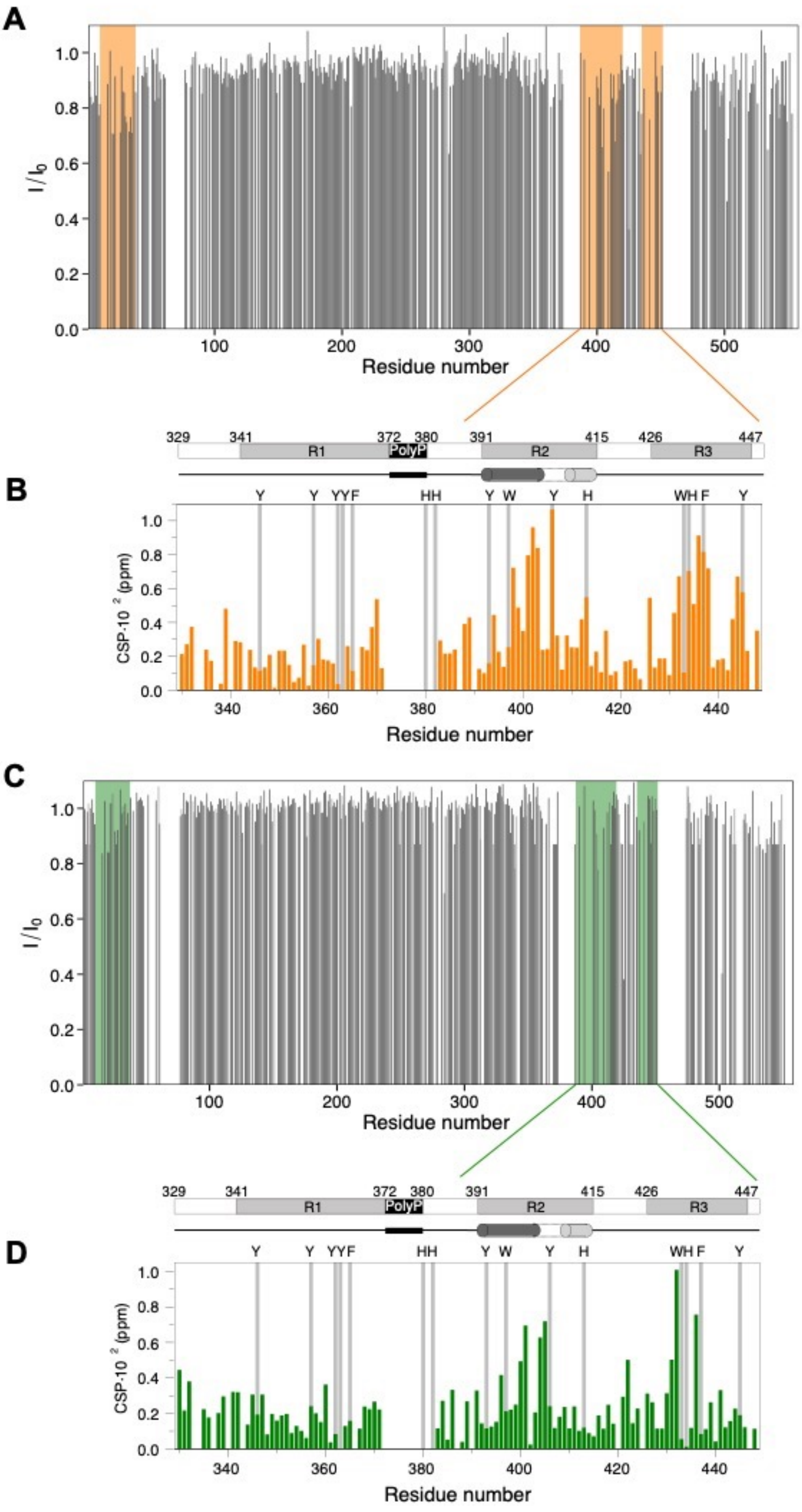

Supplementary Figure 10: Thio-2, may bind the N-terminus of the androgen receptor through a similar binding mode to EPI-001.

(A-D) Nuclear magnetic resonance (NMR) 1H-15N correlation spectra were recorded on 600 and 800 MHz Bruker Advance spectrometers equipped with cryoprobes. Experiments with 15N-labelled androgen receptor N-terminus (AR NTD) constructs NTD_{1-518} and $NTD_{330-447}$ at 25 μM were mixed with an excess amount (250 μM) of Thio-2 or EPI-001 (positive control) and measured at 5 ºC. This temperature was selected to prevent the protein to aggregate, and in consequence, affecting the resolution of the spectra. **(A and C)** Quantification of intensity changes in AR NTD (NTD_{1-518} construct) backbone amide signals in the presence of EPI-001 (A) or Thio-2 (C). **(B and D)** Quantification of chemical shift perturbations in AR NTD ($NTD_{330-447}$ construct) backbone amide signals in the presence of EPI-001 (B) or Thio-2 (D). Colored shaded boxes indicate the most affected regions.
