# Supplementary material for: Thio-2 inhibits key signaling pathways required for the development and progression of castration resistant prostate cancer
Source: Mol Cancer Ther. Author manuscript; Available in PMC 2024 Jun 5. (PMC11148553; doi:10.1158/1535-7163.MCT-23-0354)
Supplement: Figure S11 [file EMS194541-supplement-Figure_S11.pdf]

Supplementary Figure 11

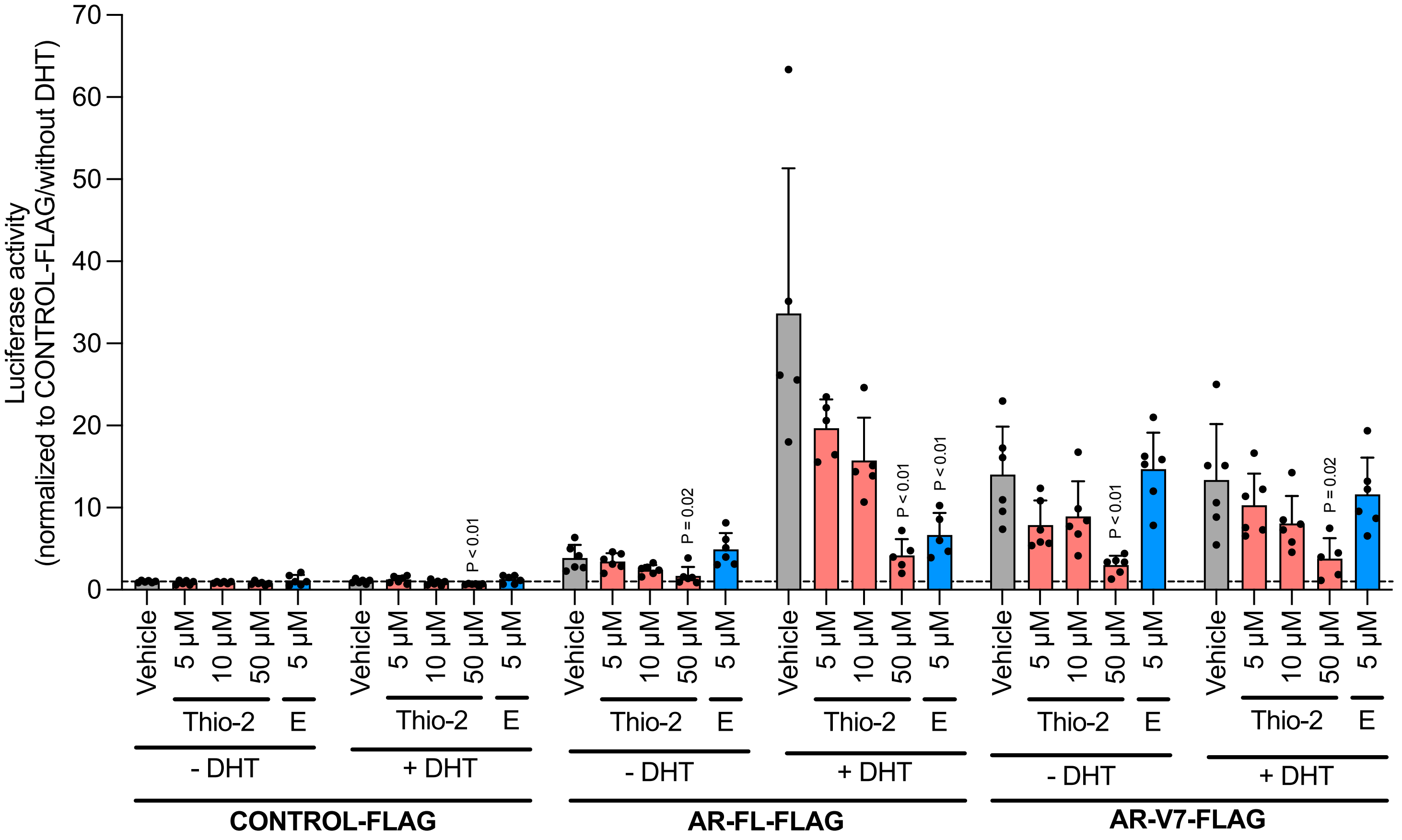

Supplementary Figure 11: Treatment with Thio-2 inhibits transactivation of the unstimulated and stimulated androgen receptor and the constitutively active androgen receptor splice variant-7.

PC3 cells were transfected with either CONTROL-FLAG, androgen receptor (AR-FL)-FLAG or AR splice variant-7 (AR-V7)-FLAG, and ARE3-PSA-luciferase (PSA-Luc), prior to treatment with either vehicle (DMSO 0.1 %), various concentrations (5, 10 or 50 μM) of Thio-2 or 5 μM enzalutamide (E) for 1 hour prior to stimulation with or without 10nM dihydrotestosterone (DHT) for 16 hours. Mean luciferase activity (compared to CONTROL-FLAG/without DHT) with standard deviation from a single experiment with five to six replicates is shown. P values were calculated for each condition compared to vehicle using unpaired Student t-test. P values ≤ 0.05 are shown.
