# Supplementary material for: Thio-2 inhibits key signaling pathways required for the development and progression of castration resistant prostate cancer
Source: Mol Cancer Ther. Author manuscript; Available in PMC 2024 Jun 5. (PMC11148553; doi:10.1158/1535-7163.MCT-23-0354)
Supplement: Figure S12 [file EMS194541-supplement-Figure_S12.pdf]

Supplementary Figure 12

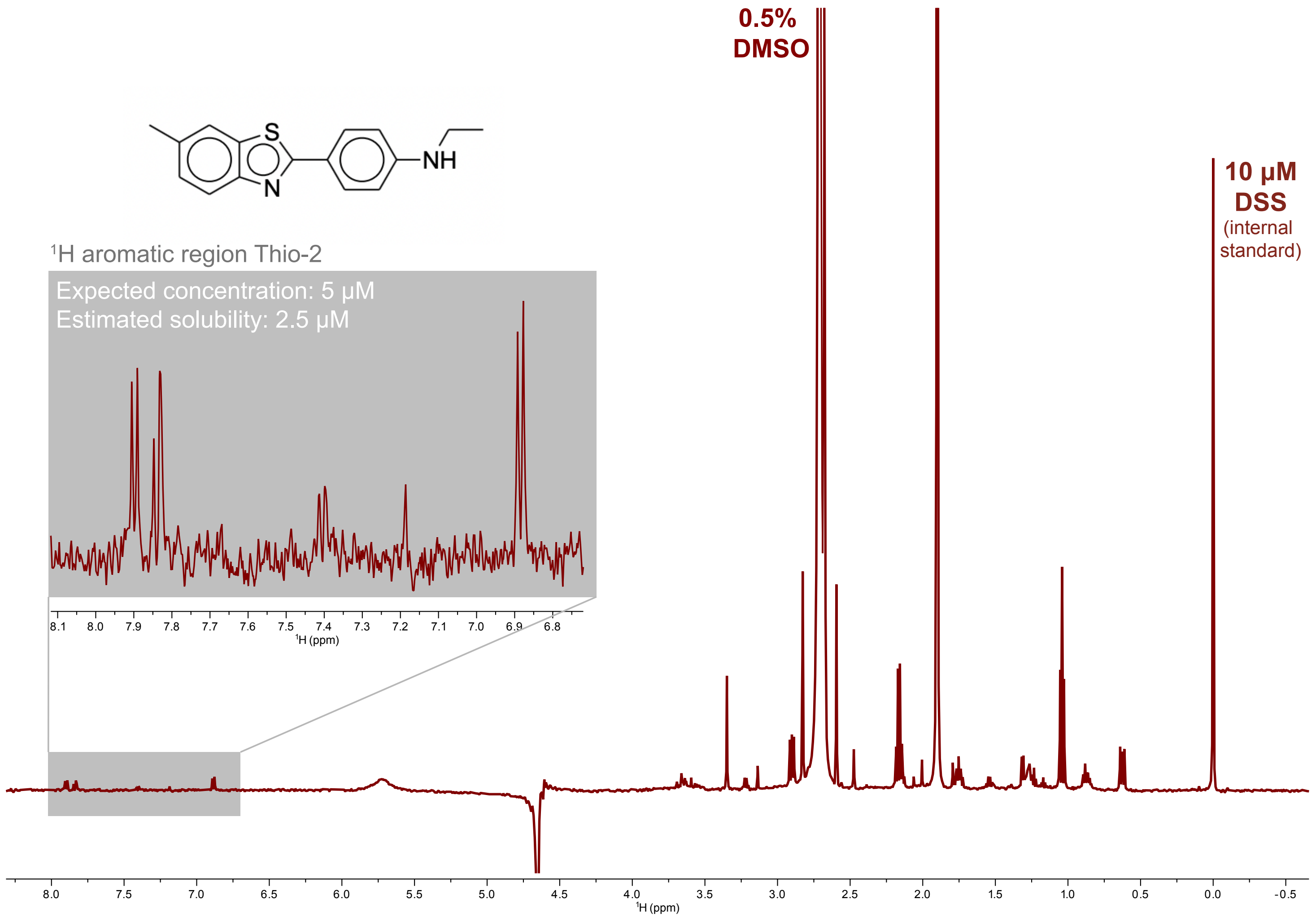

Supplementary Figure 12: Thio-2 solubility.

Thio-2 solubility was measured by integration of Thio-2 1H aromatic signals (region 6.5-8 ppm) and the internal reference (10 µM DSS) 1H signal (at 0 ppm). A sample containing 5 µM Thio-2, 10 µM DSS, and 0.5 % DMSO (buffer 20 mM sodium phosphate (pH 7.4), 1 mM TCEP, 10% $D_2 0$) were recorded on 600 MHz Bruker Avance spectrometer equipped with a cryoprobe. 1H nuclear magnetic resonance (NMR) spectra of 5 µM Thio-2 in phosphate buffer containing 0.5 % DMSO measured at 37 ºC is shown.
