# Supplementary material for: Thio-2 inhibits key signaling pathways required for the development and progression of castration resistant prostate cancer
Source: Mol Cancer Ther. Author manuscript; Available in PMC 2024 Jun 5. (PMC11148553; doi:10.1158/1535-7163.MCT-23-0354)
Supplement: Supplementary methods [file EMS194541-supplement-Supplementary_methods.docx]

**Supplementary Materials and Methods**

***In-vitro* patient derived xenograft organoid proliferation studies.**

Briefly patient derived xenograft (PDX) tumors were harvested in PDX harvesting solution (adDMEM/F12 containing 10 μM ROCK inhibitor Y27632 (Selleck Chemicals), penicillin/streptomycin, 10 mM Hepes and GlutaMAX 100× diluted all purchased form Thermofisher), cut into small pieces (< 3mm^3^) and single cell suspensions were generated by mechanical separation (40 μm Corning cell strainer, Sigma-Aldrich). Pellets were washed once on ice-cold PBS/5 mM EDTA/1x GlutaMax/10 μM Y27632, and red blood cells were removed using red blood cell lysis buffer (0.8% NH4Cl in 0.1 mM EDTA in water, buffered with KHCO3 to pH of 7.2 - 7.6, incubated 1-minute on ice) followed by another wash with ice cold PBS/5 mM EDTA/1x GlutaMax/10 μM Y27632. Single cell suspensions were either frozen for later use in BioCat BambankerTM freezing medium (Fisher Scientific) supplemented with 10 μM Y27632, or directly resuspended in ice-cold organoid growth medium (as published by Drost and colleagues with the following alterations: The p38 inhibitor SB202190 was replaced by the addition of 5 nM of NRG1) and subsequently diluted in one volume of phenol red-free, growth factor reduced, Corning MatrigelTM (Fisher Scientific) (1, 2). Organoid domes (5-50 μl) were plated as previously described by Drost and colleagues and topped up with warm medium after solidification (1). Cultures were observed over 3-7 days until visible organoid formation could be observed and then re-seeded for actual experiment. Immunohistochemistry was performed to confirm the presence of BAG-1 and AR in the developing organoids. For drug treatment, organoids were harvested in organoid harvesting solution (Amsbio), washed with medium and re-plated in the same way as described above and incubated in organoid growth medium for 5 days following drug treatment. CellTiter-Glo® 3D Cell Viability Assay (Promega) was used to assay growth of the organoids according to the manufacturer’s instruction and luminescence was measured using Synergy HTX (BioTek).

**RNA extraction.**

RNA from knockout mice was obtained by mechanical homogenization in PeqGold, RNApure solution (VWR), following the manufacturer’s instruction or, reconstituted with RNeasy RLT buffer, passed through a Qiashredder tube (Qiagen), and further processed with RNeasy Plus Mini Kit as per manufacturer's instructions. Cell line and Patient derived xenograft-organoid (PDX-O) RNA was extracted using the RNeasy Plus Mini Kit (Qiagen) as per manufacturer's instructions.

**Quantitative reverse transcription PCR (qRT-PCR).**

cDNA was synthesized using the Revertaid First Strand cDNA Synthesis kit (ThermoFisher). qRT-PCR was carried out using a ViiA 7 Real-Time PCR System (Life Technologies) using the TaqMan Universal PCR Master Mix (Applied Biosystems). TaqMan probes (ThermoFisher) used are listed in **Supplementary Table 2**. Fold change in mRNA expression levels were calculated by the comparative Ct method, using the formula 2-(-(ΔΔCt). Cell line and PDX-O samples were normalized against the average of four (GAPDH, B2M, HRPT1 and RPLP0) housekeeping genes and knockout mouse samples were normalized to mouse GAPDH.

**Western blotting.**

Cell line, patient derived xenograft (PDX) and PDX-O were lysed with RIPA buffer (Pierce^TM^; ThermoFisher) supplemented with protease inhibitor cocktail (Roche; Sigma-Aldrich) and PhosStop phosphatase inhibitor mix (Roche; Sigma-Aldrich). PDX lysate was obtained by mechanical homogenization using a Qiagen TissueLyser homogenizer according to the manufacturer’s instructions. Protein extracts (25 μg) were sonicated, heated for 5 minutes at 95C and separated on 4-12% NuPAGE® Bis-Tris gel (Invitrogen) by electrophoresis and subsequently transferred onto Immobilon-P™ PVDF membranes of 0.45 μm pore size (Millipore®; Merck). Details of primary antibodies used are provided in **Supplementary Table 3**. Chemiluminescence was detected on the Chemidoc™ Touch imaging system (Bio-Rad).

**Supplementary References**

1. Drost J, Karthaus WR, Gao D, Driehuis E, Sawyers CL, Chen Y, et al. Organoid culture systems for prostate epithelial and cancer tissue. Nat Protoc. 2016;11(2):347-58.

2. Gil V, Miranda S, Riisnaes R, Gurel B, D'Ambrosio M, Vasciaveo A, et al. HER3 Is an Actionable Target in Advanced Prostate Cancer. Cancer Res. 2021;81(24):6207-18.
