# Supplementary material for: Thio-2 inhibits key signaling pathways required for the development and progression of castration resistant prostate cancer
Source: Mol Cancer Ther. Author manuscript; Available in PMC 2024 Jun 5. (PMC11148553; doi:10.1158/1535-7163.MCT-23-0354)
Supplement: Table S1 [file EMS194541-supplement-Table_S1.docx]

| **Uniprot ID** | **PDB ID** | **Chain** | **Complex** | **Organism** | **Res** | **Method** | **Released date** |
| --- | --- | --- | --- | --- | --- | --- | --- |
| Q99933 | 1HX1 | B | HSPA8 | HUMAN | 1.90000 | X-Ray | 07/03/2001 |
| Q99933 | 3FZF | B | HSPA8 | HUMAN | 2.00000 | X-Ray | 17/03/2009 |
| Q99933 | 3FZH | B | HSPA8 | HUMAN | 1.90000 | X-Ray | 17/03/2009 |
| Q99933 | 3FZK | B | HSPA8 | HUMAN | 2.00000 | X-Ray | 17/03/2009 |
| Q99933 | 3FZL | B | HSPA8 | HUMAN | 2.00000 | X-Ray | 17/03/2009 |
| Q99933 | 3FZM | B | HSPA8 | HUMAN | 2.10000 | X-Ray | 17/03/2009 |
| Q99933 | 3LDQ | B | HSPA8 | HUMAN | 1.90000 | X-Ray | 26/01/2011 |
| Q99933 | 3M3Z | B | HSPA8 | HUMAN | 2.00000 | X-Ray | 26/01/2011 |
| Q99933 | 5AQF | D | HSPA8 | HUMAN | 1.88000 | X-Ray | 05/10/2016 |
| Q99933 | 5AQF | B | HSPA8 | HUMAN | 1.88000 | X-Ray | 05/10/2016 |
| Q99933 | 5AQG | F | HSPA8 | HUMAN | 2.24000 | X-Ray | 05/10/2016 |
| Q99933 | 5AQG | D | HSPA8 | HUMAN | 2.24000 | X-Ray | 05/10/2016 |
| Q99933 | 5AQG | B | HSPA8 | HUMAN | 2.24000 | X-Ray | 05/10/2016 |
| Q99933 | 5AQH | B | HSPA8 | HUMAN | 2.00000 | X-Ray | 05/10/2016 |
| Q99933 | 5AQI | D | HSPA8 | HUMAN | 1.98000 | X-Ray | 05/10/2016 |
| Q99933 | 5AQI | B | HSPA8 | HUMAN | 1.98000 | X-Ray | 05/10/2016 |
| Q99933 | 5AQJ | F | HSPA8 | HUMAN | 1.96000 | X-Ray | 05/10/2016 |
| Q99933 | 5AQJ | D | HSPA8 | HUMAN | 1.96000 | X-Ray | 05/10/2016 |
| Q99933 | 5AQJ | B | HSPA8 | HUMAN | 1.96000 | X-Ray | 05/10/2016 |
| Q99933 | 5AQK | B | HSPA8 | HUMAN | 2.09000 | X-Ray | 05/10/2016 |
| Q99933 | 5AQL | D | HSPA8 | HUMAN | 1.69000 | X-Ray | 05/10/2016 |
| Q99933 | 5AQL | B | HSPA8 | HUMAN | 1.69000 | X-Ray | 05/10/2016 |
| Q99933 | 5AQM | D | HSPA8 | HUMAN | 1.63000 | X-Ray | 05/10/2016 |
| Q99933 | 5AQM | B | HSPA8 | HUMAN | 1.63000 | X-Ray | 05/10/2016 |
| Q99933 | 5AQN | B | HSPA8 | HUMAN | 2.45000 | X-Ray | 05/10/2016 |
| Q99933 | 5AQN | F | HSPA8 | HUMAN | 2.45000 | X-Ray | 05/10/2016 |
| Q99933 | 5AQN | D | HSPA8 | HUMAN | 2.45000 | X-Ray | 05/10/2016 |
| Q99933 | 5AQO | F | HSPA8 | HUMAN | 2.12000 | X-Ray | 05/10/2016 |
| Q99933 | 5AQO | D | HSPA8 | HUMAN | 2.12000 | X-Ray | 05/10/2016 |
| Q99933 | 5AQO | B | HSPA8 | HUMAN | 2.12000 | X-Ray | 05/10/2016 |
| Q99933 | 5AQP | F | HSPA8 | HUMAN | 2.08000 | X-Ray | 05/10/2016 |
| Q99933 | 5AQP | D | HSPA8 | HUMAN | 2.08000 | X-Ray | 05/10/2016 |
| Q99933 | 5AQP | B | HSPA8 | HUMAN | 2.08000 | X-Ray | 05/10/2016 |
| Q99933 | 5AQQ | F | HSPA8 | HUMAN | 2.72000 | X-Ray | 05/10/2016 |
| Q99933 | 5AQQ | D | HSPA8 | HUMAN | 2.72000 | X-Ray | 05/10/2016 |
| Q99933 | 5AQQ | B | HSPA8 | HUMAN | 2.72000 | X-Ray | 05/10/2016 |
| Q99933 | 5AQR | F | HSPA8 | HUMAN | 1.91000 | X-Ray | 05/10/2016 |
| Q99933 | 5AQR | D | HSPA8 | HUMAN | 1.91000 | X-Ray | 05/10/2016 |
| Q99933 | 5AQR | B | HSPA8 | HUMAN | 1.91000 | X-Ray | 05/10/2016 |
| Q99933 | 5AQS | D | HSPA8 | HUMAN | 2.00000 | X-Ray | 05/10/2016 |
| Q99933 | 5AQS | B | HSPA8 | HUMAN | 2.00000 | X-Ray | 05/10/2016 |
| Q99933 | 5AQT | B | HSPA8 | HUMAN | 1.90000 | X-Ray | 05/10/2016 |
| Q99933 | 5AQU | B | HSPA8 | HUMAN | 1.92000 | X-Ray | 05/10/2016 |
| Q99933 | 5AQV | B | HSPA8 | HUMAN | 1.75000 | X-Ray | 05/10/2016 |

**Supplementary Table 1: List of the 44 3D structures of HSC70 (HSPA8) in complex with the BAG domain of BAG-1 used in comparative structural analyses.**

ID – Identifier, PDB – protein data bank, Res – resolution, X-Ray – X-Ray diffraction.
