# Supplementary material for: Thio-2 inhibits key signaling pathways required for the development and progression of castration resistant prostate cancer
Source: Mol Cancer Ther. Author manuscript; Available in PMC 2024 Jun 5. (PMC11148553; doi:10.1158/1535-7163.MCT-23-0354)
Supplement: Table S2 [file EMS194541-supplement-Table_S2.docx]

.

| **Gene target** | **Company** | **Catalogue ID** |
| --- | --- | --- |
| mouse BAG-1 exon 1/2 | ThermoFisher | Mm01208597_g1 |
| mouse BAG-1 exon 5/6 | ThermoFisher | Mm01208593_m1 |
| mouse BAG-1 exon 6/7 | ThermoFisher | Mm00437765_m1 |
| mouse GAPDH | ThermoFisher | Mm99999915_m1 |
| human PSA | ThermoFisher | Hs02576345_m1 |
| human TMPRSS2 | ThermoFisher | Hs05024838_m1 |
| human FKBP5 | ThermoFisher | Hs01561006_m1 |
| human GAPDH | ThermoFisher | Hs02786624_g1 |
| human B2M | ThermoFisher | Hs00187842_m1 |
| human HRPT1 | ThermoFisher | Hs00363810_m1 |
| human RPLP0 | ThermoFisher | Hs00420895_gH |

**Supplementary Table 2:** **TaqMan probes for qRT-PCR analyses**
