# Supplementary material for: Thio-2 inhibits key signaling pathways required for the development and progression of castration resistant prostate cancer
Source: Mol Cancer Ther. Author manuscript; Available in PMC 2024 Jun 5. (PMC11148553; doi:10.1158/1535-7163.MCT-23-0354)
Supplement: Table S3 [file EMS194541-supplement-Table_S3.docx]

| **Protein target** | **Species (clone)** | **Company** | **Catalogue ID/RRID** |
| --- | --- | --- | --- |
| panmoBAG-1 | mouse  (AF815) | R&D systems | AF815/AB_2062286 |
| GAPDH | mouse/human  (G9) | Santa Cruz | sc-365062/AB_10847862 |
| panBAG-1 | human  (RM356) | RevMAb | 31-1242-00/AB_2783595 |
| AR-FL | human (D6F11) | Cell Signaling | 5153/AB_10691711 |
| AR-V7 | human  (RM7) | RevMAb | 31-1109-00/AB_2716436 |
| PSA | human (D11E1) | Cell Signaling | 2475/AB_2797601 |
| Vinculin | mouse/human  (hVIN-1) | Sigma-Aldrich | V9131/AB_477629 |
| AR-FL | mouse/human  (EPR1535(2)) | abcam | ab133273/AB_11156085 |
| Beta-actin | mouse/human  (C4) | Santa Cruz | sc-47778/AB_626632 |
| C-MYC | mouse/rat/human (Y69) | abcam | ab32072/AB_731658 |

**Supplementary Table 3:** **Antibodies used for western blot analyses**

pan-mouse-BAG-1 – panmoBAG-1, pan-BAG-1 – panBAG-1.
