# Supplementary material for: Thio-2 inhibits key signaling pathways required for the development and progression of castration resistant prostate cancer
Source: Mol Cancer Ther. Author manuscript; Available in PMC 2024 Jun 5. (PMC11148553; doi:10.1158/1535-7163.MCT-23-0354)
Supplement: Table S4 [file EMS194541-supplement-Table_S4.docx]

| **Protein target (species, clone, supplier, RRID)** | **Retrieval buffer**  **(method)** | **Dilution**  **(time)** | **Detection** | **Controls** |
| --- | --- | --- | --- | --- |
| panmoBAG-1 (mouse, AF815, R&D systems, AB_2062286) | pH6 ER1 30min  (Leica Biosystems Bond RX) | 1:500  (1 hour)  Rabbit anti-goat IgG secondary (Abcam) 1:5000 (30min) | Leica Novolink Polymer Detection System | siBAG-1 (neg) and siControl (pos) NIH 3T3 |
| AR-FL (mouse/human, EPR1535(2), abcam, AB_11156085) | pH6 citrate (pressure cooker) | 1:500 (1 hour) | DAKO EnVision Detection System | siAR (neg) and siControl (pos) TRAMPC2, mouse normal prostate |
| panBAG-1 (human, RM356, RevMAb, AB_2783595) | pH8.1 Tris/EDTA  (microwave) | 1:1500  (1 hour) | DAKO EnVision Detection System | siBAG-1 (neg) and siControl (pos) HeLa; SKMEL2 (pos) |

**Supplementary Table 4:** **Protocols and antibodies for immunohistochemical analyses**

pan-mouse-BAG-1 – panmoBAG-1, pan-BAG-1 – panBAG-1, pos – positive, neg – negative.
