# Supplementary material for: Thio-2 inhibits key signaling pathways required for the development and progression of castration resistant prostate cancer
Source: Mol Cancer Ther. Author manuscript; Available in PMC 2024 Jun 5. (PMC11148553; doi:10.1158/1535-7163.MCT-23-0354)
Supplement: Table S5 [file EMS194541-supplement-Table_S5.docx]

| **Cell line** | **Supplier** | **Catalogue ID/RRID** | **Media^** |
| --- | --- | --- | --- |
| 22Rv1 | ATCC | CRL-2505/CVCL_1045 | RMPI1640/10% FBS |
| LNCaP | ATCC | CRL-1740/CVCL_1379 | RMPI1640/10% FBS |
| LNCaP95 | Dr Meeker/Dr Luo* | CVCL_ZC87 | RMPI 1640^^/10%CCS |
| NIH3T3 | ATCC | CRL-1658/CVCL_0594 | DMEM/10% FBS |
| HeLa | ATCC | CCL-2/CVCL_0030 | DMEM/10% FBS |
| TRAMPC2 | ATCC | CRL-2731/CVCL_3615 | DMEM/10% FCS |
| PC3 | ATCC | CRL-1435/CVCL-0035 | RMPI1640/10% FBS |
| DU145 | ATCC | HTB-81/CVCL_0105 | DMEM/10% FCS |

**Supplementary Table 5: Cell lines**

ATCC – American Type Culture Collection, FBS – fetal bovine serum, CSS – charcoal stripped serum, * - LNCaP95 cells were a kind gift from Drs. Alan K Meeker and Jun Luo (Johns Hopkins University, Baltimore, Maryland, USA), ^ - supplementation as per supplier instructions, ^^ - phenol red free.
