# Supplementary material for: Thio-2 inhibits key signaling pathways required for the development and progression of castration resistant prostate cancer
Source: Mol Cancer Ther. Author manuscript; Available in PMC 2024 Jun 5. (PMC11148553; doi:10.1158/1535-7163.MCT-23-0354)
Supplement: Table S6 [file EMS194541-supplement-Table_S6.docx]

| **Gene target** | **Supplier** | **Catalogue ID** |
| --- | --- | --- |
| human/mouse control | Dharmacon (Horizon) | D-001810-10-05 |
| human BAG-1 | Dharmacon (Horizon) | L-003871-00-0005 |
| mouse BAG-1 | Dharmacon (Horizon) | L-042153-00-0005 |
| mouse AR-FL | Dharmacon (Horizon) | L-050296-00-0005 |
| human MYC | Dharmacon (Horizon) | L-003282-02-0005 |

**Supplementary Table 6: ON-TARGETplus siRNA pools for gene expression knockdown**
