# Supplementary material for: Thio-2 inhibits key signaling pathways required for the development and progression of castration resistant prostate cancer
Source: Mol Cancer Ther. Author manuscript; Available in PMC 2024 Jun 5. (PMC11148553; doi:10.1158/1535-7163.MCT-23-0354)
Supplement: Table S7 [file EMS194541-supplement-Table_S7.docx]

| **Guide** | **sgRNA** | **Sequence** |
| --- | --- | --- |
| BAG-1 g1 | CRISPRevolution EZ RNA-1 (BAG1+33262829) | A*G*G*UCGUGCUUCUCAUUG*C*C* |
| BAG-1 g2 | CRISPRevolution EZ RNA-2 (BAG1+33262809) | C*C*U*GCUGGGAGGUAACAU*G*A* |
| BAG-1 g3 | CRISPRevolution EZ RNA-3 (BAG1+33262794) | C*U*G*GUUCACUGCUGCCCU*G*C* |
| BAG-1 g4 | CRISPRevolution EZRNA-4 (BAG1+33262768) | U*G*A*ACCAGUUGUCCAAGA*C*C* |

**Supplementary Table 7: Synthego BAG-1 sgRNA for BAG-1 CRISPR knockout clones**
